# Multi-Omics and Single-Cell Dissection of Exostosin Glycosyltransferases (EXT1/EXT2) Reveals Divergent Oncogenic Roles and Therapeutic Vulnerabilities in Gliomas

**DOI:** 10.7150/jca.123965

**Published:** 2026-01-01

**Authors:** Yi-Chun Chiang, Chih-Yang Wang, Neethu Palekkode, Shun-Fa Yang, Kai-Fu Chang, Ching-Chung Ko, Chih-Hsuan Chang, Hui-Ru Lin, Chi-Jen Wu, Yu-Cheng Ho, Chih-Chun Lin, Chien-Han Yuan, Sachin Kumar, Dahlak Daniel Solomon, Juan Lorell Ngadio, Fitria Sari Wulandari, Do Thi Minh Xuan, Chung-Bao Hsieh, Meng-Chi Yen, I-Jeng Yeh, Pi-Chan Ko, Chia-Lung Shih, Hoi-Bor Chan, Yung-Kuo Lee, Ngoc Uyen Nhi Nguyen

**Affiliations:** 1 Institute of Medicine, Chung Shan Medical University, Taichung 40201, Taiwan.; 2 Department of Surgery, Division of Neurosurgery, Kaohsiung Armed Forces General Hospital, Kaohsiung 80284, Taiwan.; 3 Graduate Institute of Cancer Biology and Drug Discovery, College of Medical Science and Technology, Taipei Medical University, Taipei 11031, Taiwan.; 4 PhD Program for Cancer Molecular Biology and Drug Discovery, College of Medical Science and Technology, Taipei Medical University, Taipei 11031, Taiwan.; 5 Department of Biotechnology, Mother Teresa Women's University, Kodaikanal, Tamil Nadu, 624101, India.; 6 Department of Medical Research, Chung Shan Medical University Hospital, Taichung 40201, Taiwan.; 7 Medical Laboratory, Medical Education and Research Center, Kaohsiung Armed Forces General Hospital, Kaohsiung 80284, Taiwan.; 8 Division of Experimental Surgery Center, Department of Surgery, Tri-Service General Hospital, National Defense Medical University, Taipei 11490, Taiwan.; 9 Department of Medical Imaging, Chi-Mei Medical Center, Tainan710402, Taiwan.; 10 Department of Health and Nutrition, Chia Nan University of Pharmacy and Science, Tainan 71710, Taiwan.; 11 School of Medicine, College of Medicine, National Sun Yat-Sen University, Kaohsiung 80424, Taiwan.; 12 Institute of Medical Science and Technology, National Sun Yat-Sen University, Kaohsiung 80424, Taiwan.; 13 Nursing Department, Kaohsiung Armed Forces General Hospital, Kaohsiung 80284, Taiwan.; 14 College of Nursing, Kaohsiung Medical University, Kaohsiung 80708, Taiwan.; 15 School of Medicine, College of Medicine, I-Shou University, Kaohsiung 82445, Taiwan.; 16 Department of Physical Therapy, I-Shou University, Kaohsiung 824005, Taiwan.; 17 Department of Otolaryngology, Kaohsiung Armed Forces General Hospital, Kaohsiung 80284, Taiwan.; 18 Department of Otolaryngology, National Defense Medical University, Taipei 11490, Taiwan.; 19 Faculty of Applied Sciences and Biotechnology, Shoolini University of Biotechnology and Management Sciences, Himachal Pradesh 173229, India.; 20 Yogananda School of AI Computers and Data Sciences, Shoolini University, Solan 173229, India.; 21 Department of Bioinformatics, School of Life Sciences, Indonesia International Institute for Life Sciences, Jl. Pulomas Barat Kav 88, Jakarta Timur 13210, Indonesia.; 22 Faculty of Pharmacy, Van Lang University, 69/68 Dang Thuy Tram Street, Binh Loi Trung Ward, Ho Chi Minh City, 70000, Vietnam.; 23 Division of General Surgery, Department of Surgery, Tri-Service General Hospital, Taipei 114202, Taiwan.; 24 Department of Emergency Medicine, Kaohsiung Medical University Hospital, Kaohsiung Medical University, Kaohsiung 80708, Taiwan.; 25 Graduate Institute of Clinical Medicine, College of Medicine, Kaohsiung Medical University, Kaohsiung 80708, Taiwan.; 26 Department of Neurosurgery, Ditmanson Medical Foundation Chiayi Christian Hospital, Chiayi 60002, Taiwan.; 27 Clinical Research Center, Ditmanson Medical Foundation Chiayi Christian Hospital, Chiayi City 60002, Taiwan.; 28 School of Medicine, National Defense Medical University, Taipei 11490, Taiwan.; 29 Center for Regenerative Medicine, University of South Florida Health Heart Institute, Tampa, Florida 33602, USA.; 30 Division of Cardiology, Department of Internal Medicine, Morsani School of Medicine, University of South Florida, Tampa, Florida 33602, USA.

**Keywords:** Glycosylation, Exostosin Glycosyltransferase 1 (EXT1), Exostosin Glycosyltransferase 2 (EXT2), Glioma, Multi-Omics, Biomarker

## Abstract

Exostosin glycosyltransferase 1 (*EXT1*) and exostosin glycosyltransferase 2 (*EXT2*) catalyze heparan sulfate chain elongation and are increasingly implicated in cancer biology, but their roles in gliomas remain incompletely defined. Here, we performed an integrative multi-omics analysis to dissect the transcriptional, epigenetic, and microenvironmental landscape of *EXT1* and *EXT2* across gliomas. Bulk transcriptomic data from The Cancer Genome Atlas (TCGA) and the Chinese Glioma Genome Atlas (CGGA) revealed that both EXT1 and EXT2 are upregulated in high-grade gliomas and associate with adverse survival, with EXT1 showing the strongest and most consistent prognostic impact. Gene set enrichment analysis (GSEA) and gene set variation analysis (GSVA) indicated that EXT1-high tumors are enriched for DNA damage and replication stress programs, cell cycle progression, inflammatory response, and stromal activation pathways, whereas EXT2 expression is preferentially linked to extracellular matrix remodeling, cytoskeletal organization and angiogenesis-related signaling. Single-cell RNA sequencing and Immune deconvolution using Cell-type Identification By Estimating Relative Subsets Of RNA Transcripts (CIBERSORT) and Estimation of STromal and Immune cells in MAlignant Tumor tissues using Expression data (ESTIMATE) showed that *EXT1* correlates with increased stromal and immune scores, and reduced cytotoxic T cell signatures, consistent with an immunosuppressive tumor microenvironment. *EXT2* expression is enriched in gliomas with pronounced vascular and mesenchymal features, supporting a complementary role in invasive growth and tissue remodeling. Immunohistochemistry on a glioma tissue microarray validated the upregulation of *EXT1* protein in high-grade tumors. The study findings identified *EXT1* as a central glycosylation-linked regulator of replication stress tolerance and immune remodeling in gliomas, and suggest that *EXT2* contributes to extracellular matrix and cytoskeletal reprogramming. The exostosin axis represents a promising source of prognostic biomarkers and potential therapeutic targets in glioma.

## 1. Introduction

Gliomas are the most prevalent and clinically challenging primary brain tumors in adults, comprising a heterogeneous group of malignancies that range from low-grade gliomas (LGG) to the highly aggressive glioblastoma multiforme (GBM) 1. Among them, GBM is the most lethal subtype, marked by pronounced intratumoral heterogeneity, rapid progression, and poor responses to standard therapies. Despite advances in neurosurgery, radiotherapy, and temozolomide-based chemotherapy, the median survival of GBM patients remains dismal, rarely exceeding 15 months 2-4. This persistent therapeutic failure is largely attributed to the molecular and cellular complexity of gliomas, including transcriptional subtypes, therapy-resistant stem-like cell populations, and an immunosuppressive tumor microenvironment (TME).

Recent studies emphasized the role of post-translational modifications such as glycosylation, along with epigenetic reprogramming and cell-type-specific regulatory programs, in shaping gliomas' pathobiology 5. Among these, glycosylation, a tightly regulated enzymatic process essential for proteoglycan biosynthesis, and extracellular matrix (ECM) remodeling has gained particular attention. The exostosin family of glycosyltransferases, including *EXT1* and *EXT2* 6, 7, catalyzes the polymerization of heparan sulfate (HS) chains, which in turn modulate cell signaling, growth factor availability, and immune surveillance 8, 9. While inactivating mutations in *EXT1/2* are well-documented in hereditary exostoses 10, their functional roles in cancer 11, 12, and in gliomas specifically, remain poorly defined. Pan-cancer analyses suggested context-dependent oncogenic or tumor-suppressive functions for *EXT1* and *EXT2*, but a systematic investigation of their transcriptional, epigenetic, and functional states in gliomas is lacking 13, 14. The emerging large-scale analyses have further highlighted the growing importance of glycosylation in glioma biology 15, 16. A study based on glycosylation-related gene signatures (GRMSs) identified an independent prognostic factor across TCGA, CGGA, and Rembrandt glioma cohorts, demonstrating that aberrant glycosylation strongly influences the tumor grade, immune infiltration, and overall survival (OS) 17-21. Similarly, large-scale transcriptomic surveys revealed that glycosylation pathway genes are coordinately dysregulated across multiple cancers, positioning glycosyltransferases as central mediators of metabolic and structural adaptation in the TME 22-24. However, most existing studies have treated glycosylation as a broad transcriptional signature rather than dissecting the functional contributions of individual glycosyltransferases. In particular, the upstream enzymes that initiate HS chain synthesis, such as *EXT1* and *EXT2*, have not been systematically evaluated in gliomas 25, 26.

**Figure 1:**
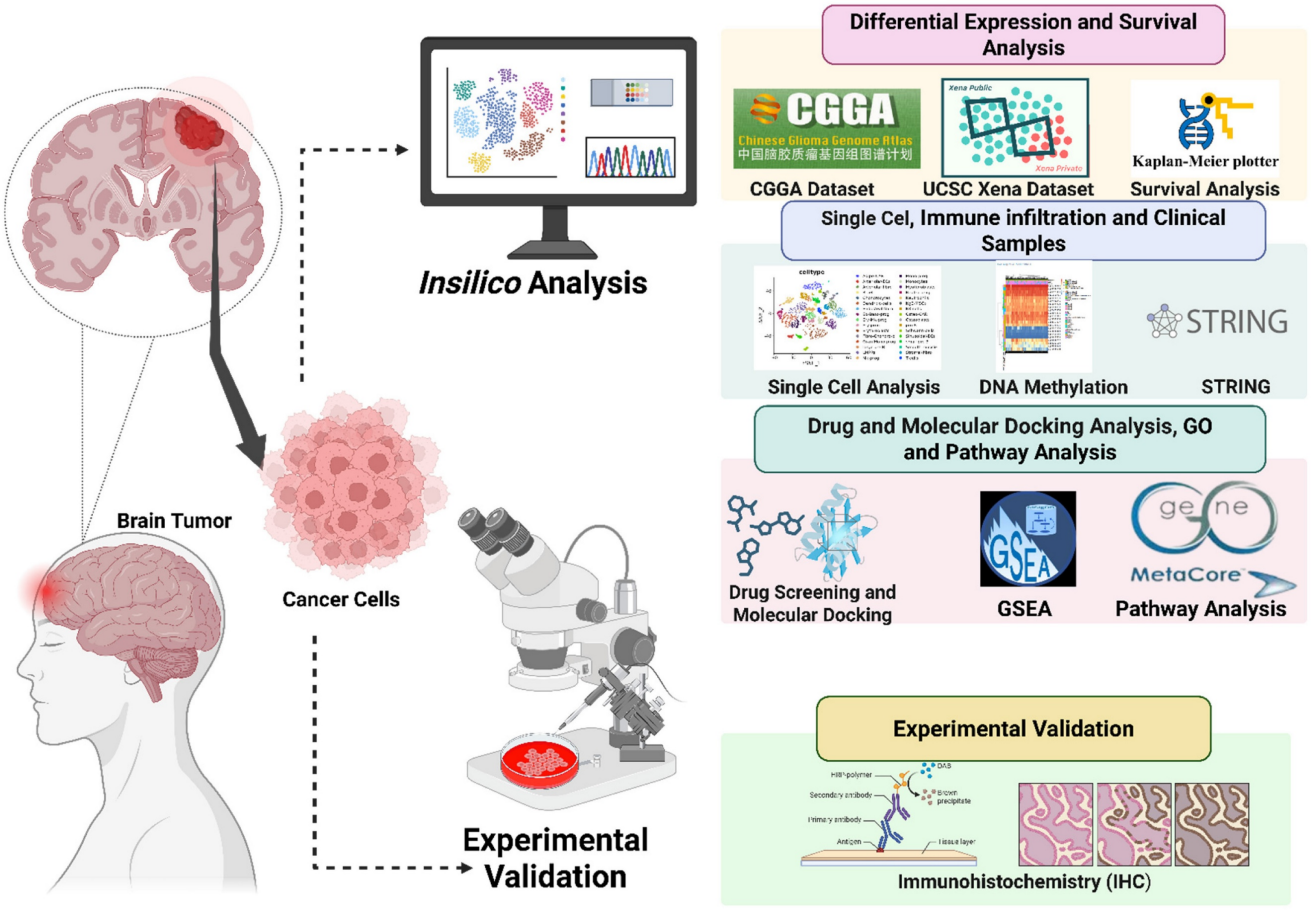
** Schematic workflow of integrated *in silico* and* in vitro* analyses of *EXT1/EXT2* in gliomas**.

Previous work largely focused on downstream heparinase or sulfatase 2 (SULF2)-mediated extracellular remodeling, leaving a major gap in our understanding of how core HS-polymerizing enzymes regulate tumor cell signaling, spatial heterogeneity, and therapeutic resistance 27. Moreover, single-cell atlases that capture spatial distributions of glycosylation enzymes in gliomas have not yet been fully integrated with DNA methylation or pharmacogenomic data. By addressing these questions, our study provides one of the first molecular and spatially resolved characterizations of the *EXT1/2* axis in gliomas. Beyond elucidating the mechanism, this integrative approach defines a glycosylation-centered landscape that can guide the development of glycosyltransferase-based biomarkers and precision therapeutic strategies for patient stratification in malignant gliomas (Fig. 1).

## 2. Materials and Methods

### 2.1 Data Acquisition, Preprocessing, and Gene Expression Analysis

In this study, multiple publicly available datasets were integrated to comprehensively analyze *EXT1* and *EXT2* expressions, epigenetic regulation, and pathway associations in human gliomas. Bulk RNA-sequencing (RNA-seq) expression profiles, corresponding clinical annotations, and OS data were obtained from TCGA through the UCSC Xena browser (https://xenabrowser.net/), and from the CGGA database (http://www.cgga.org.cn/) 28, 29. Normal brain tissue transcriptomic data were retrieved from the GTEx project (https://gtexportal.org/), which provides reference expression profiles from non-diseased tissues. The GEPIA web platform (http://gepia.cancer-pku.cn/) was used for initial pan-cancer comparisons of *EXT1* and *EXT2* expressions between tumor and normal tissues 30. GEPIA integrates TCGA and GTEx datasets under a unified processing pipeline, allowing differential expression analyses using normalized transcript per million (TPM) values and log₂(TPM+1) transformation 31-33. Box plots were generated through GEPIA's visualization interface to assess differential expression across cancer types, including gliomas. Survival analyses were performed using both CGGA and TCGA glioma cohorts to evaluate the prognostic significance of *EXT1* and *EXT2* expressions. Normalized RNA-seq and clinical survival data were obtained from the CGGA portal, which hosts harmonized CGGA datasets 29. In addition, the GlioVis portal (https://gliovis.bioinfo.cnio.es/) was used as an independent validation interface to visualize expression and survival relationships of *EXT1* and *EXT2* across multiple glioma datasets, including TCGA-GBM, TCGA-LGG, and TCGA-GBM/LGG datasets, ensuring consistency across independent cohorts 34. For both datasets, gene expression values were log2(TPM+1) transformed, and patients were dichotomized into high- and low-expression groups based on median expression levels of *EXT1* and *EXT2* 35-37. Kaplan-Meier survival curves were generated using the survival and survminer R packages, and log-rank tests were applied to assess statistical significance. Hazard ratios (HRs) and 95% confidence intervals (CIs) were calculated using univariate Cox proportional hazards models 38-40.

### 2.2 Protein-Protein Interaction (PPI) Network and DNA Methylation Analysis

To explore functional associations of *EXT1*- and *EXT2*-related gene networks, we used the STRING database (https://www.string-db.org/) to construct PPI networks. Genes strongly co-expressed with *EXT1* or *EXT2* in TCGA glioma datasets were uploaded to STRING, and networks were generated using a confidence score threshold of 0.7 (high confidence). Network visualizations highlighted hub genes based on degree of centrality and functional clustering, providing insights into biological pathways potentially regulated by *EXT1* and *EXT2* 41. For the DNA methylation analysis, we used MethSurv (https://biit.cs.ut.ee/methsurv/) to assess the prognostic significance of cytosine-phosphate-guanine (CpG) methylation sites within *EXT1* and *EXT2* loci. Illumina HumanMethylation450K beta values from TCGA glioma samples were queried, and individual CpG sites were evaluated for their association with OS using univariate Cox proportional hazards models. CpG sites showing strong negative correlations with gene expression were prioritized, consistent with potential transcriptional repression. Heatmaps and survival plots were generated directly from MethSurv, and results were integrated with our expression and network analyses to characterize the epigenetic regulation of *EXT1* and *EXT2* in gliomas 42.

### 2.3 Single cell RNA-sequencing (scRNA-seq)

The scRNA-seq datasets of human gliomas (GSE182109) were obtained from the Gene Expression Omnibus (GEO), which together provide comprehensive coverage of glioblastoma and lower-grade glioma specimens with annotated cellular identities. Expression matrices and accompanying metadata were downloaded as provided by the original studies. All analyses were performed in R (v4.2.2) using the Seurat package (v4.3.0). Cells were filtered to remove low-quality profiles, retaining those with 200-7000 detected genes and <10% mitochondrial gene content. Data normalization was conducted using Seurat's NormalizeData function with a scale factor of 10,000, followed by log-transformation (log1p) and scaling with ScaleData to standardize expression across genes 43-45. The top 2000 variable genes were selected using the “vst” method implemented in FindVariableFeatures. To minimize technical variability between datasets and enable direct comparisons, we applied Seurat's integration workflow (FindIntegrationAnchors and IntegrateData) using canonical correlation analysis (CCA). Integration was performed using the top 30 principal components (dims = 1:30) to align shared biological features while reducing dataset-specific noise. Dimensionality reduction and visualization were conducted using Uniform Manifold Approximation and Projection (UMAP), and clusters were annotated based on canonical lineage markers to identify glioma cells, oligodendrocyte precursor (OPC)-like cells, astrocytic-like cells, mesenchymal-like cells, myeloid cells (microglia/macrophages), T and B lymphocytes, endothelial cells, and pericytes. These annotations were cross-validated with the original metadata to ensure consistent labeling across cohorts 46, 47. Expression patterns of *EXT1* and *EXT2* were visualized using Seurat's FeaturePlot, VlnPlot, and DotPlot functions across cell-type clusters. All visualizations were generated using standardized color scales and log-normalized expression values to ensure comparability between datasets 48, 49.

### 2.4. Comprehensive Gene Set Enrichment (GSEA) and Pathway Analyses

To investigate the functional pathways linked to *EXT1* and *EXT2* expressions in gliomas, we performed a GSEA using the clusterProfiler package in R (v4.2.2). Processed RNA-seq expression data for glioma patients were obtained from TCGA-LGG and TCGA-GBM cohorts via the UCSC Xena browser. Samples were divided into *EXT1*-high/low and *EXT2*-high/low groups according to their respective median expression levels. An enrichment analysis was conducted against the Molecular Signatures Database (MSigDB v7.5), including Hallmark gene sets. Ranked gene lists were generated based on log2 fold-change (FC) values from a differential expression analysis, and enrichment scores were computed using the preranked method with 1000 permutations. Pathways meeting the thresholds of an FDR q-value of < 0.25 and a nominal p-value of < 0.05 were considered significant 50, 51. To complement the GSEA results, MetaCore (Clarivate Analytics) was used for functional annotation and signaling pathway mapping 52-54. For this analysis, the top 10% of *EXT1*- and *EXT2*-correlated genes (Spearman |ρ| ≥ 0.6, FDR < 0.001) were identified from TCGA datasets, and pathway enrichment was performed in MetaCore to identify significantly overrepresented biological processes and molecular networks 55-57. Statistical significance was defined as p < 0.05, and results were visualized using bar plots, enrichment maps, and network diagrams to highlight key pathways associated with *EXT1* and *EXT2* in gliomas 58-62.

### 2.5. Drug Sensitivity Analysis and Molecular Docking

Expression-drug sensitivity correlations were evaluated via GSCA using Genomics of Drug Sensitivity in Cancer (GDSC) and Clinical Trials Reporting Program (CTRP) datasets 63, 64. EXT1/2 expressions were correlated with 50% inhibitory concentration (IC_50_) values. Docetaxel, bleomycin, and tanespimycin (17-N-allylamino-17-demethoxygeldanamycin (17-AAG)) were selected for modeling. To further validate EXT1/2's roles, molecular docking was conducted to predict interactions with small-molecule inhibitors 65-67. 17-AAG, bleomycin, and docetaxel were selected based on the GSCA and CTRP analyses and validated using molecular docking methods 26-28. The SDF structure file of 17-AAG was retrieved from PubChem (https://pubchem.ncbi.nlm.nih.gov/) while SMILES values of bleomycin and docetaxel were converted to PDB using NovoPro (https://www.novoprolabs.com/tools/smiles2pdb). Ligand preprocessing was performed using PyMol and AutoDockTools prior to docking. Protein structures of *EXT1/2* were taken from RCSB PDB (https://www.rcsb.org/) using the structure code 7SCJ. Chain A of the structure was used, representing the *EXT1/EXT2* complex, and was cleaned using standard preprocessing workflows in PyMol and AutoDockTools. The binding site was determined using uridine-5'-diphosphate (UDP) as the main binding location according to the PDB entry. Docking was then performed using Vina with an energy range of 4 and an exhaustiveness setting of 8. Three-dimensional and two-dimensional visualizations of the docked complexes were generated using PyMol and LigPlot+ 68, 69.

### 2.6. Clinical Tissue Microarray (TMA) and Immunohistochemistry (IHC)

Formalin-fixed paraffin-embedded (FFPE) samples from gliomas patients (n = 52) were collected from Kaohsiung Armed Forces General Hospital with Institutional Review Board (IRB) approval (KAFGHIRB 113-050), comprising World Health Organization (WHO) grade II (n = 20), III (n = 16), IV (n = 16) gliomas, and five adjacent normal brain tissues. Representative tumor cores (1.5 mm) were arrayed into TMAs by Hao-Long Biotechnology-Ltd., Kaohsiung City, Taiwan. Sections (4 μm) were deparaffinized and rehydrated, followed by citrate buffer-based antigen retrieval. *EXT1* staining was performed using anti-EXT1 (HPA044394, 1:200, Merck) and horseradish peroxidase (HRP)-based DAB detection (Vector Labs), with hematoxylin counterstaining. Slides were scanned (Leica AT2), and *EXT1* intensity was quantified using QuPath v0.3.2 based on the H-score formula = ∑(i × Pi), where i = staining intensity (0-3) and Pi = percentage of cells. Interobserver agreement was high (κ > 0.90). Statistical analyses were performed using one-way analysis of variance (ANOVA) followed by Tukey's post-hoc test 70-72.

### 2.7 Statistical Analysis

The statistical analyses were performed using R (v4.1.0). Kaplan-Meier survival curves were plotted, and the log-rank test was used for survival comparisons. Cox regression analysis evaluated HRs for *EXT1/2* expression levels in relation to LGG patient survival. Spearman's correlation was used to examine the relationship between *EXT1/2* expressions and DNA methylation. A p-value < 0.05 was considered statistically significant.

## 3. Results

### 3.1 *EXT1/2* Are Differentially Expressed Across Cancers and Prioritized in Gliomas

**Figure 2:**
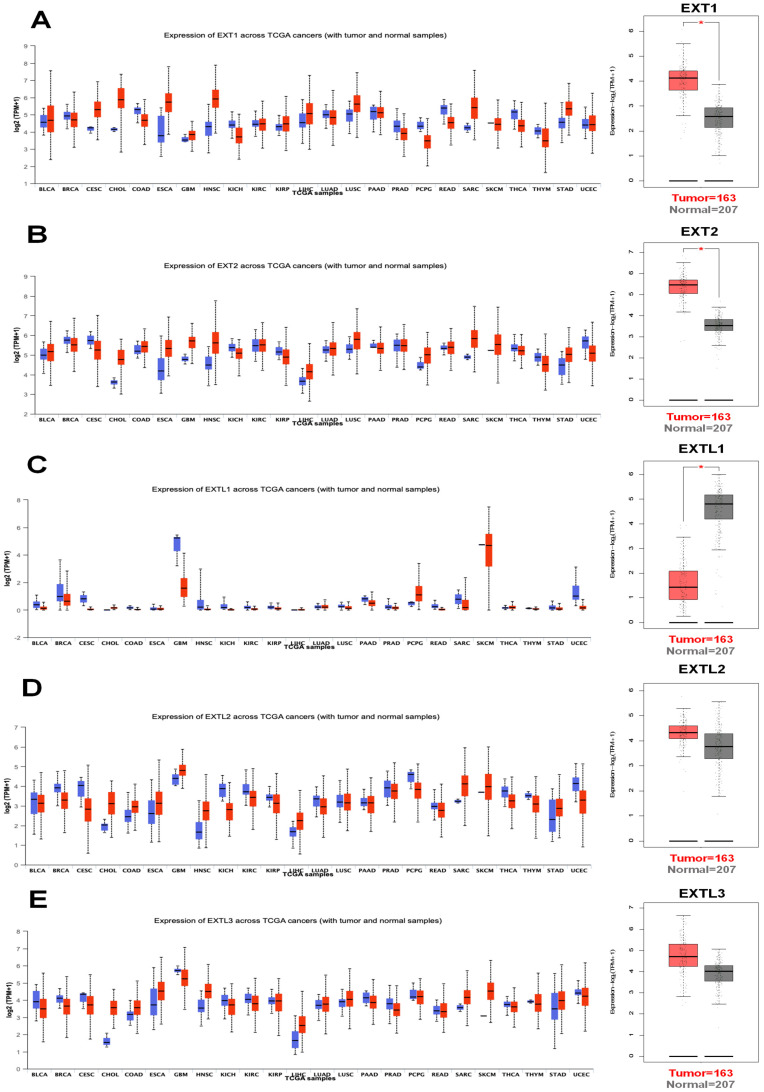
** Pan-cancer expression profiles of *EXT* family genes with emphasis on gliomas.** Boxplots display mRNA expression levels of (A) *EXT1*, (B) *EXT2*, (C) *EXTL1*, (D) *EXTL2*, and (E) *EXTL3* across 33 tumor types, including low-grade gliomas (LGG), using transcriptomic data from TCGA and GTEx. Expression values are presented as log₂(TPM + 1). Tumor and matched normal tissues are respectively shown in red and blue boxes. Numbers of tumor (T) and normal (N) samples for each cancer type are indicated below each plot. Statistical comparisons were performed using Student's *t*-test; asterisks (*) denote significance at *p* < 0.05.

To identify glycosyltransferases relevant to gliomas, we conducted a pan-cancer analysis of EXT family genes (*EXT1, EXT2,* and* EXTL1-3*) using harmonized TCGA and GTEx transcriptomic datasets. Among the 33 tumor types analyzed, *EXT1* and *EXT2* were significantly upregulated in all primary glioma samples relative to normal brain samples (Fig. 2A, B), a pattern similarly observed in several other tumor types. Conversely, *EXTL1, EXTL2,* and *EXTL3* showed inconsistent and context-specific expression patterns across cancers (Fig. 2C-E). Given their consistent dysregulation and biological relevance, EXT1 and EXT2 were selected as the principal isoforms for downstream multi-omics analyses in gliomas. To further confirm their glioma-specific transcriptional upregulation, we performed a differential expression analysis between glioma and normal brain samples from the TCGA cohort (Supplementary Fig. S1A). Both *EXT1* and* EXT2* appeared among the significantly upregulated genes (log_2_fold change (FC) > 1.5, adjusted p < 0.001), underscoring their potential oncogenic activation.

### 3.2 *EXT1* and *EXT2* Stratify Clinical Subtypes and Predict Survival of Glioma Patients

**Figure 3:**
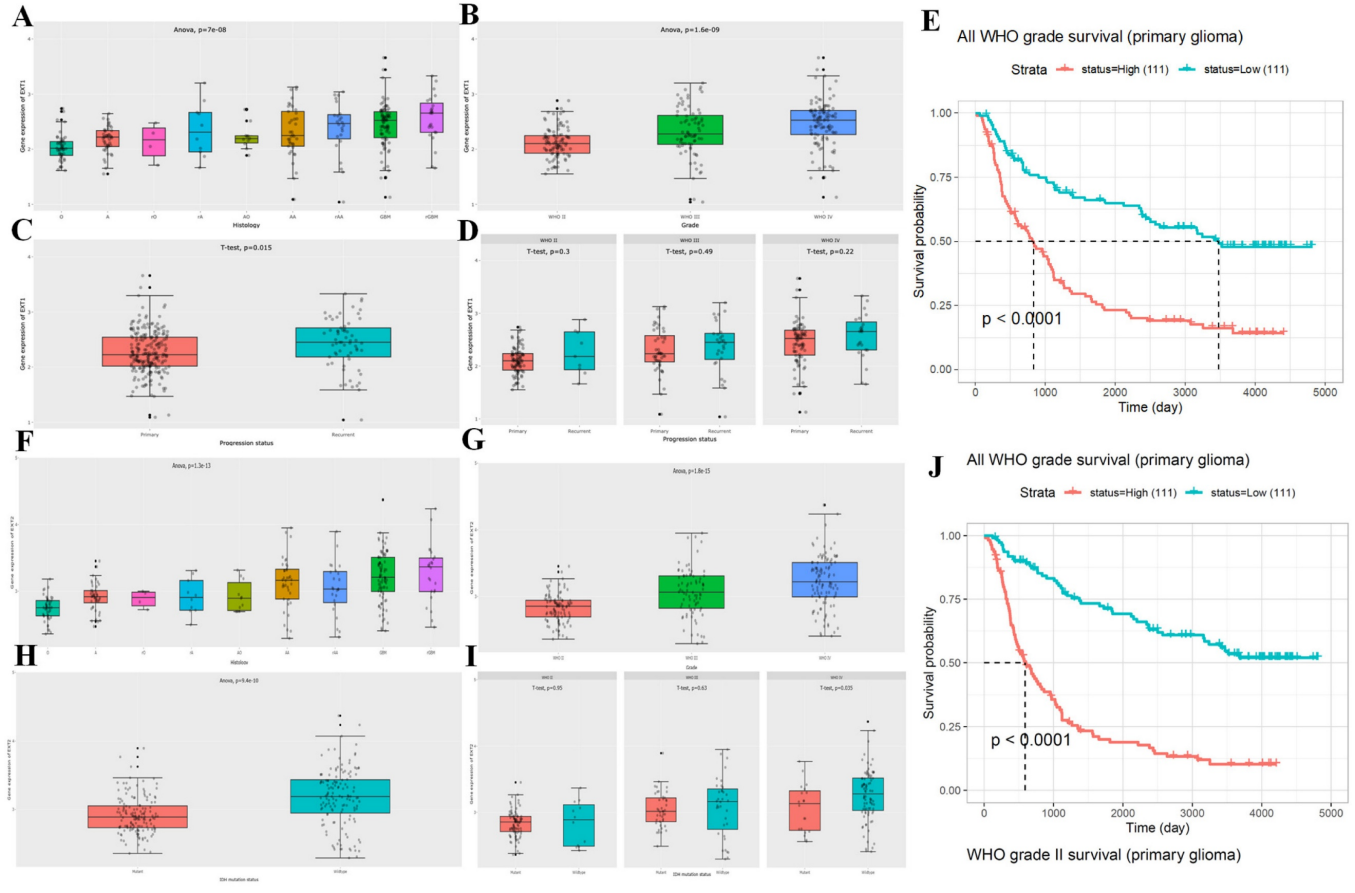
** Clinical and prognostic correlations of *EXT1* and *EXT2* expressions in primary glioma patients across WHO grades.** Boxplots (A-D, F-I) and Kaplan-Meier survival curves (E, J) illustrate the clinical relevance of *EXT1* and *EXT2* expression in gliomas. (A) *EXT1* expression differs significantly among histological subtypes (ANOVA, p < 7 × 10⁻⁸), with higher levels in more aggressive phenotypes such as GBM. (B) *EXT1* expression increases with advancing WHO grade (II-IV; ANOVA, p < 1.6 × 10⁻⁹). (C) Overall, recurrent gliomas show significantly higher *EXT1* expression than primary tumors (t-test, p = 0.015). (D) When stratified by WHO grade II, III, and IV, *EXT1* expression does not differ significantly between primary and recurrent tumors (all t-tests p > 0.2), suggesting that the difference in panel C is largely driven by grade composition. (E) High *EXT1* expression is associated with significantly worse overall survival in primary glioma across all WHO grades (log-rank p < 0.0001). (F) *EXT2* expression varies significantly across histological subtypes (ANOVA, p < 1 × 10⁻¹³), with higher levels in more aggressive glioma phenotypes. (G) *EXT2* levels increase with higher WHO grades (ANOVA, p < 1 × 10⁻¹⁵). (H) *EXT2* expression is significantly higher in IDH-wildtype than in IDH-mutant gliomas (ANOVA, p < 4 × 10⁻¹⁰). (I) Within WHO grades II, III, and IV, *EXT2* expression does not differ significantly between IDH-mutant and IDH-wildtype tumors (all t-tests p > 0.05), indicating that the strong association in panel H is mainly driven by grade distribution. (J) Kaplan-Meier survival analysis shows that high *EXT2* expression predicts poorer overall survival specifically in WHO grade II primary gliomas (log-rank p < 0.0001).

We then analyzed the expression patterns of *EXT1* and *EXT2* across clinical and molecular subtypes in the CGGA glioma patient cohort. *EXT1* expression significantly varied by WHO grade, with higher levels observed in high grade gliomas (Fig. 3A, B). Additionally, *EXT1* was modestly upregulated in recurrent compared to primary tumors and showed a positive correlation with tumor progression metrics (Fig. 3C, D). A Kaplan-Meier analysis revealed that patients with high *EXT1* expression had markedly shorter OS, with the strongest prognostic discrimination in WHO II/III tumors (Fig. 3E). In contrast, *EXT2* displayed a similar but less robust pattern across glioma subtypes (Fig. 3F-I), showing modest elevation in lower grades but no significant difference between primary and recurrent tumors. Survival analysis indicated that *EXT2*-high expression was associated with poorer outcomes (Fig. 3J). To further validate these survival trends, we extended the analysis to an independent public dataset (Supplementary Fig. S1B, C). These findings position *EXT1* as a stronger and more consistent prognostic biomarker, particularly in lower-grade gliomas, and suggest that *EXT1* may aid clinical risk stratification beyond existing molecular classifiers.

### 3.3. *EXT1* and *EXT2* Exhibit Distinct Protein Interaction Networks and Epigenetic in Gliomas

**Figure 4:**
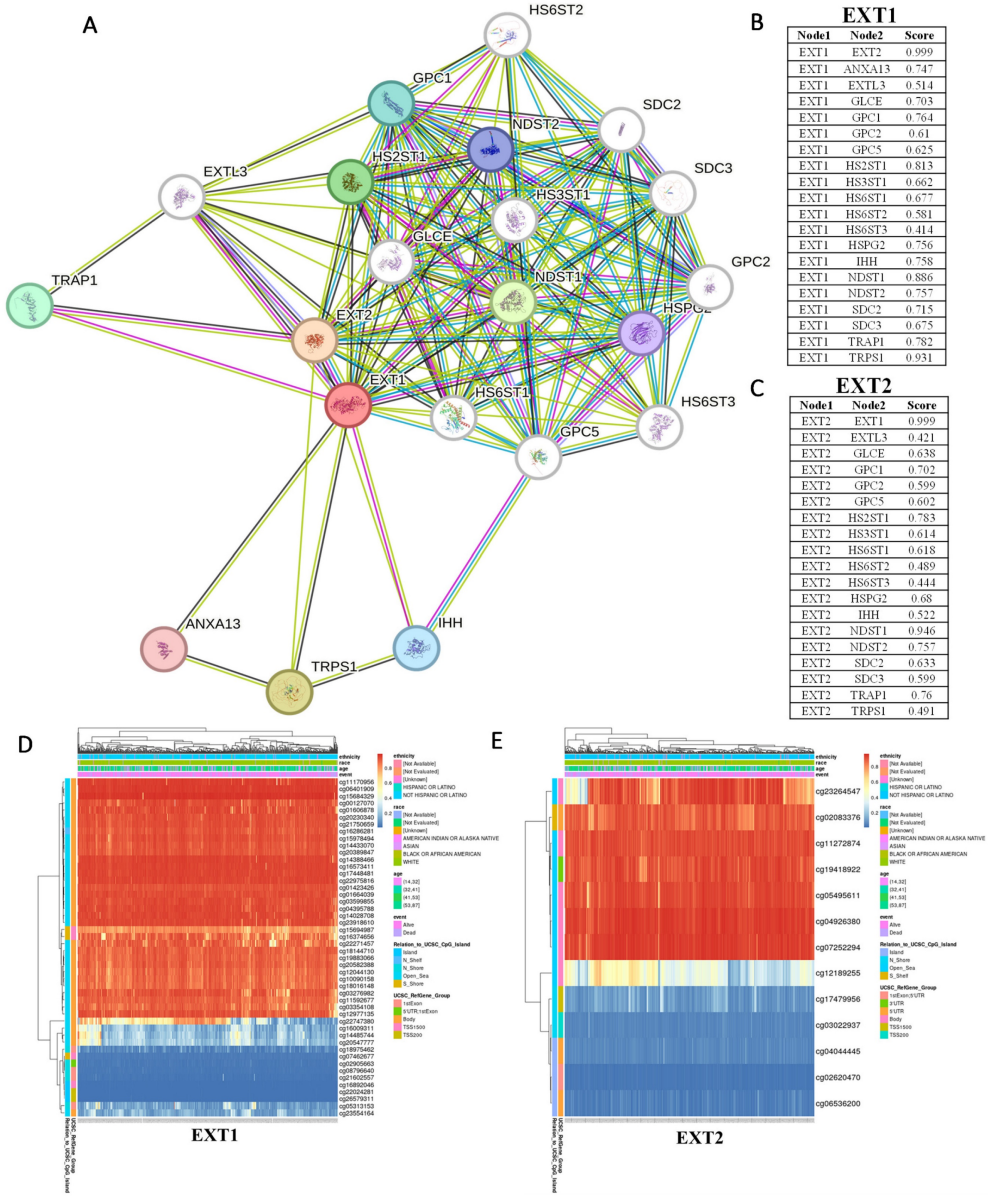
** Protein-protein interaction (PPI) networks and DNA methylation profiles of *EXT1* and *EXT2* in gliomas.** (A-C) STRING-based PPI networks highlight interaction partners of *EXT1* (red node) and *EXT2* (orange node). The line thickness corresponds to interaction confidence scores. Right panels show ranked confidence values for top *EXT1*- and *EXT2*-interacting proteins. (D, E) Heatmap visualization of DNA methylation profiles for *EXT1* and *EXT2* loci across TCGA glioma patients. Unsupervised clustering identified hypomethylated CpG clusters (blue) as being strongly correlated with increased *EXT1/2* expressions (red), supporting epigenetic de-repression as a mechanism. Clinical covariates (WHO grade, IDH status, and 1p/19q codeletion) are annotated above each heatmap.

To further elucidate the molecular contexts of *EXT1* and* EXT2*, we constructed PPI networks using STRING based on their top co-expressed genes in gliomas. Both *EXT1* and* EXT2* were embedded within the HS biosynthetic machinery, clustering tightly with N-deacetylase/N-sulfotransferase 1/2 (NDST1/2), HS 5-O-sulfotransferase 1/2 (HS6ST1/2), D-glucuronyl C5-epimerase (GLCE), and glypican family members, highlighting their conserved roles in HS chain elongation. Beyond this shared enzymatic module, however, the networks revealed distinct interaction biases. *EXT1* exhibited strong associations with tumor necrosis factor receptor-associated protein 1 (*TRAP1*) and other stress-adaptive regulators, suggesting a role in replication stress buffering and checkpoint fidelity, which aligns with our transcriptomic analyses implicating *EXT1* in ATR/checkpoint kinase 1 (*CHK1*) signaling. By contrast, *EXT2* preferentially interacted with glypicans and syndecans, reinforcing its involvement in cell adhesion and cytoskeletal remodeling, consistent with its enrichment in mesenchymal-like glioma cells and Ras homology (Rho) guanosine triphosphatase (GTPase)-driven invasive programs. These findings support the idea that *EXT1* and* EXT2* occupy non-redundant yet complementary hubs within the glioma interactome, contributing both to replication-stress adaptation in stromal compartments and to tumor-intrinsic invasion (Fig. 4A-C). To further investigate upstream mechanisms driving their dysregulation, we next analyzed the DNA methylation landscapes of *EXT1* and *EXT2* in TCGA gliomas (Fig. 4D, E). Hierarchical clustering of CpG probes revealed recurrent hypomethylated clusters within both genes, particularly at promoter and gene-body regulatory sites.

### 3.4. Single-Cell Transcriptomic Profiling Reveals Compartmentalized Expressions of *EXT1* and *EXT2* in Gliomas

**Figure 5:**
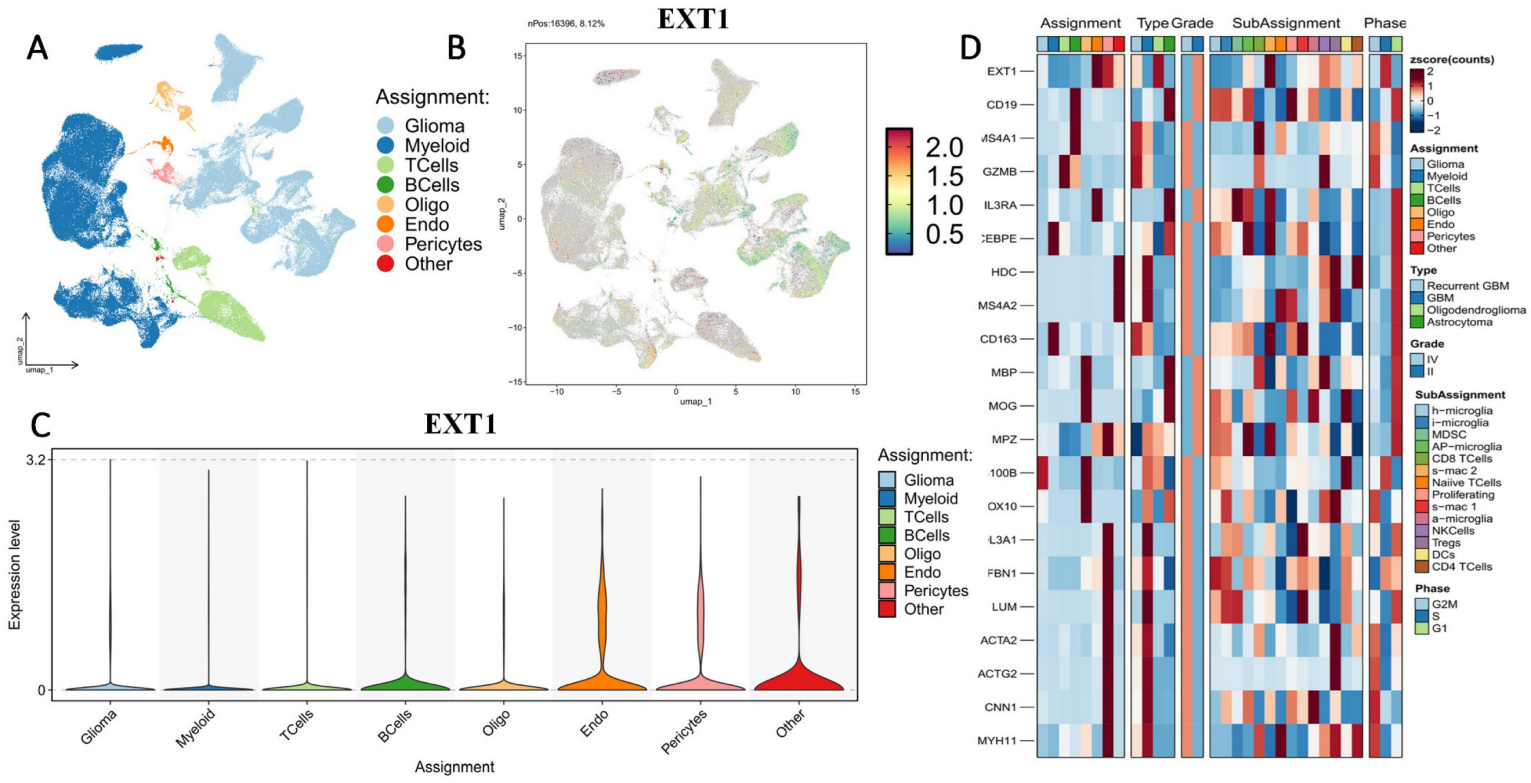
** Single-cell transcriptomic profiling of *EXT1* in gliomas.** (A) UMAP plot showing major cell-type clusters, including gliomas, myeloid cells, T cells, B cells, oligodendrocytes (Oligo), endothelial cells (Endo), pericytes, and others. (B) UMAP feature plot showing the expression distribution of *EXT1* across all cells. (C) Violin plot of *EXT1* expression levels in each cell type. (D) Heatmap showing scaled expression (z-score) of *EXT1* and representative marker genes across different cell-type assignments, tumor types, WHO grades, immune subpopulations, and cell cycle phases.

**Figure 6:**
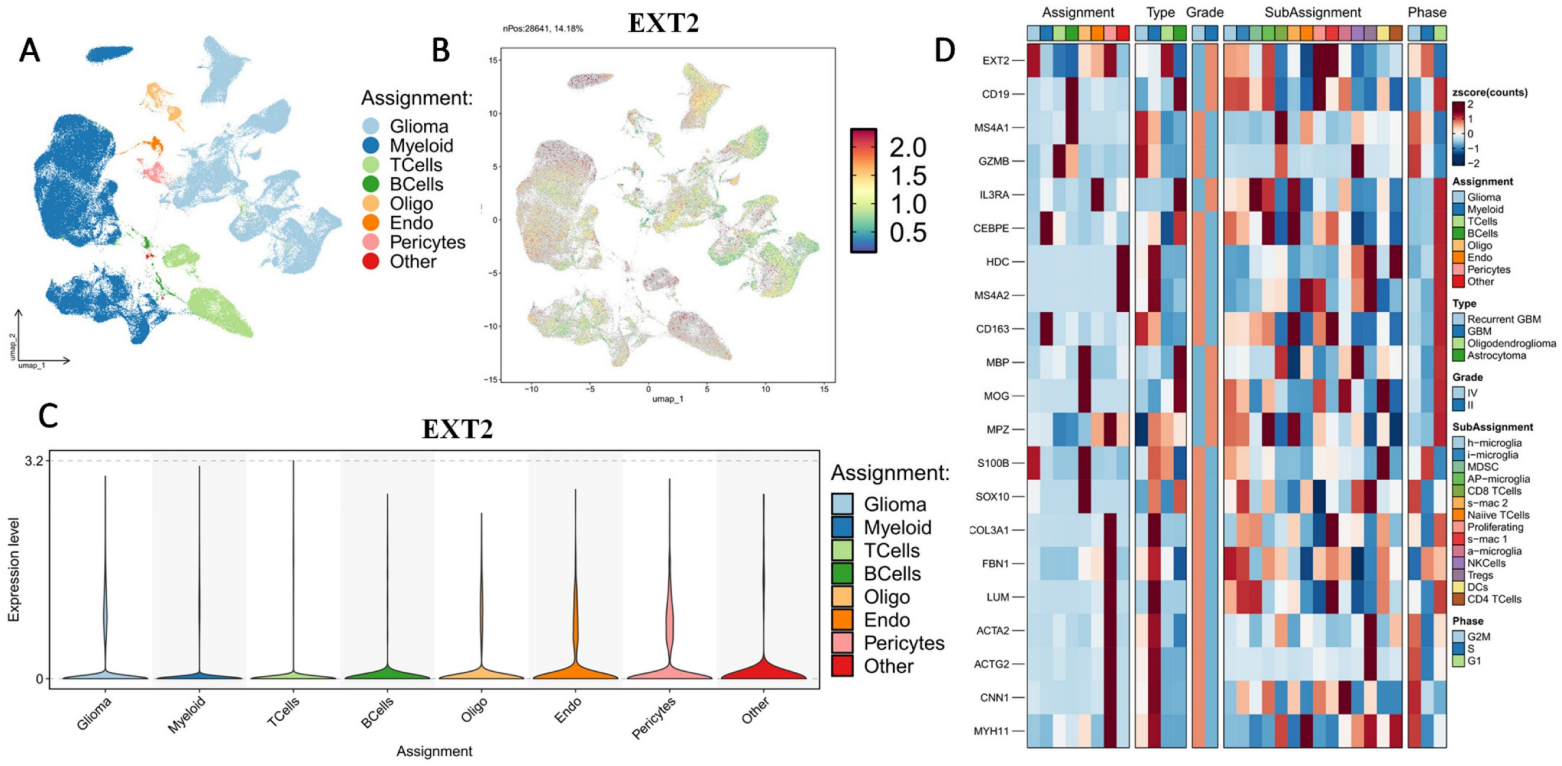
** Single-cell transcriptomic profiling of *EXT2* in gliomas.** (A) UMAP plot showing major cell-type clusters, including glioma, myeloid cells, T cells, B cells, oligodendrocytes (Oligo), endothelial cells (Endo), pericytes, and others. (B) UMAP feature plot showing the expression distribution of *EXT2* across all cells. (C) Violin plot of *EXT2* expression levels in each cell type. (D) Heatmap showing scaled expression (z-score) of *EXT2* and representative marker genes across different cell-type assignments, tumor types, WHO grades, immune subpopulations, and the cell cycle.

**Figure 7:**
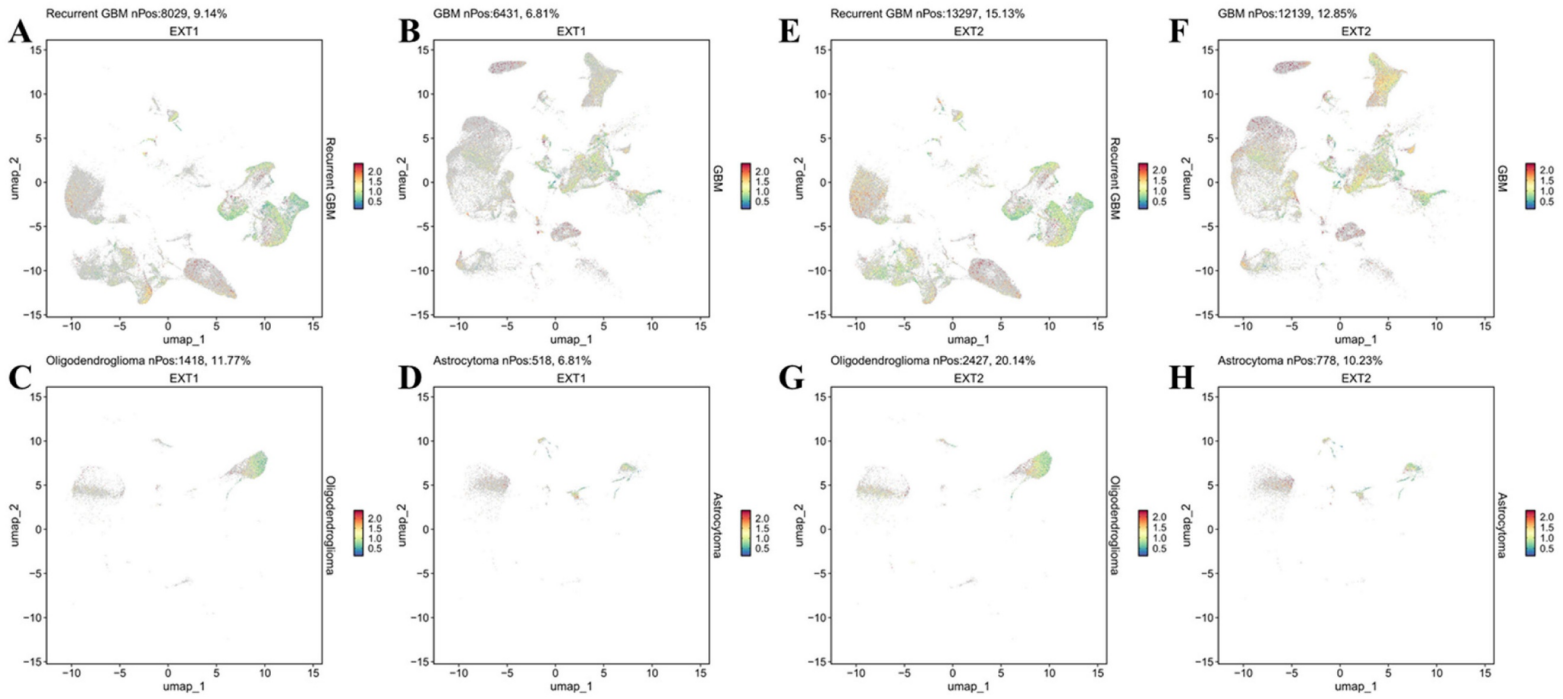
** Histology-specific single-cell expression patterns of *EXT1* and *EXT2* in GBM.** (A-D) UMAP feature plots showing normalized *EXT1* expression in GBM, oligodendroglioma, and astrocytoma. (E-H) Corresponding UMAP feature plots for *EXT2* expression in the same histologic subtypes. Each dot represents an individual cell colored by scaled gene expression (blue = low, red = high). “nPos” indicates the number of cells within that tumor type with expression above the detection threshold, and the percentage (%) denotes their proportion relative to the total number of cells in that histology.

First, we examined the cell-type-specific distribution of *EXT1*. The UMAP projection revealed that EXT1 expression was highly concentrated in endothelial and pericyte populations, with moderate expression observed in OPC-like glia (Fig. 5). In Supplementary Figure S2, *EXT1* expression was predominantly confined to vascular-stromal compartments, with detectable transcripts in 10,975 glioma cells (13.4%), 3473 myeloid cells (3.8%), and markedly higher proportions of endothelial (28.9%) and pericyte (27.2%) clusters. Expression in lymphoid subsets, including T and B cells, remained negligible (<8 %). This distribution pattern suggests that *EXT1* aligns with endothelial cells (forming blood vessels) and stromal cells (supporting connective tissue), indicating a role in maintaining ECM integrity and microvascular structure. Because *EXT1* encodes a key HS polymerase, its enrichment in vascular territories supports the hypothesis that it contributes to angiogenesis and endothelial-tumor communication. Moreover, these perivascular territories are known to buffer replication stress and foster immune exclusion. The co-expression of *EXT1* with stromal markers such as *ACTA2*, lumican (*LUM*), and *COL3A1* suggests that it may promote myofibroblast-like states, reinforcing basement membrane deposition and limiting cytotoxic immune infiltration. Figure 5 thus aligns well with our earlier findings that *EXT1* participates in replication-stress adaptation and stromal niche conditioning, highlighting its supportive role in the TME. Next, we analyzed expression patterns of *EXT2* across single-cell clusters (Fig. 6). In contrast to *EXT1, EXT2* was predominantly expressed in malignant glioma cell populations, particularly those enriched in mesenchymal and contractile gene signatures. *EXT2*-high clusters co-expressed *COL3A1, FBN1, ACTA2, CNN1,* and *MYH11*, indicating activation of cytoskeletal and motility-related pathways. These features are consistent with a mesenchymal-like glioma phenotype, in which cytoskeletal-remodeling and focal-adhesion signaling enable enhanced traction generation and cell migration. The presence of *EXT2* in these aggressive cell states implies a role in mechanical adaptability and resistance to mitotic or DNA-damaging stress, which aligns with our pharmacogenomic results showing reduced drug sensitivity in *EXT2*-high tumors. Supplementary Figure S3 extends these observations by comparing *EXT1* and *EXT2* expressions across histologic subtypes, genomic states, and tumor grades. Both genes were markedly upregulated in recurrent and high-grade gliomas (WHO III and IV), confirming their progressive activation during malignant transformation. *EXT1* and *EXT2* were particularly enriched in recurrent GBM and oligodendrogliomas, consistent with *EXT1*'s vascular-stromal localization and *EXT2*'s mesenchymal distribution (Fig. 7).

### 3.5 *EXT1* and *EXT2* Drive Distinct Oncogenic Programs as Revealed by a GSEA

**Figure 8:**
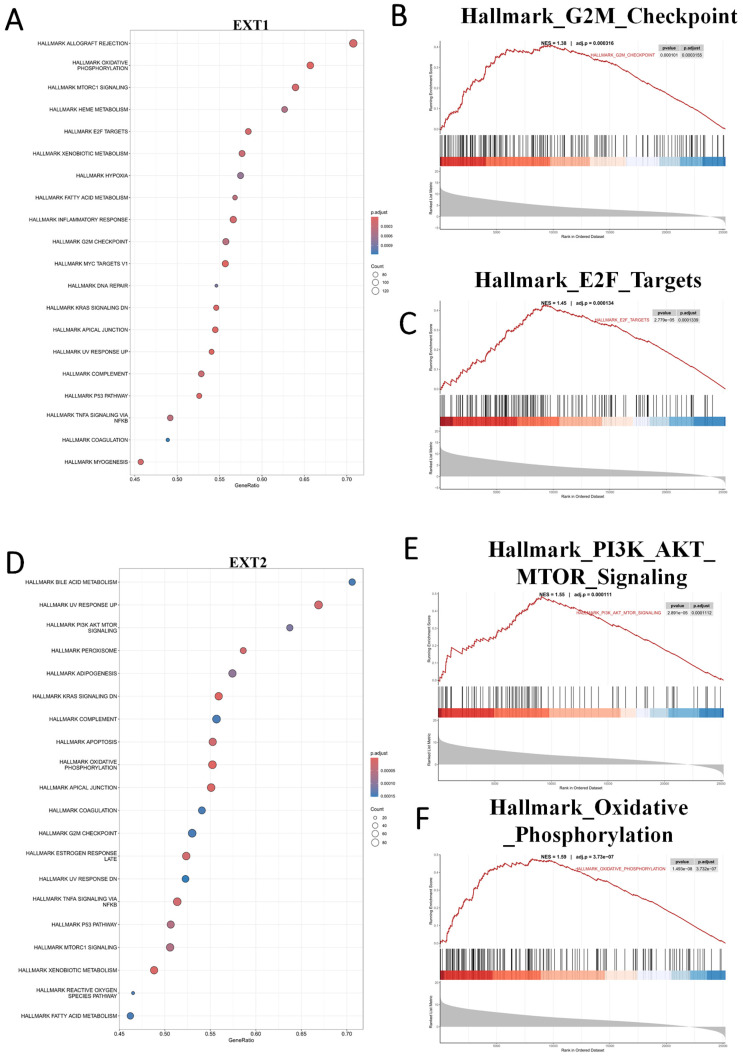
** Gene set enrichment analysis (GSEA) reveals distinct oncogenic programs associated with *EXT1* and *EXT2* in gliomas.** (A) Dot plot summarizing MSigDB Hallmark gene sets positively enriched in *EXT1*-high tumors. (B, C) Representative GSEA enrichment plots demonstrating upregulation of the Hallmark G2M checkpoint and Hallmark E2F targets signatures in *EXT1*-high samples. (D) Dot plot summarizing MSigDB Hallmark gene sets positively enriched in EXT2-high tumors. (E,F) Representative GSEA enrichment plots demonstrating PI3K/AKT/MTOR signaling and the Hallmark oxidative phosphorylation signature in *EXT2*-high samples.

To further characterize the divergent oncogenic roles of *EXT1* and* EXT2*, we performed a GSEA on glioma cohorts stratified by high versus low expression of each gene. *EXT1* was significantly enriched in pathways related to DNA repair and replication stress adaptation, including ATR/CHK1 checkpoint signaling, E2F target activation, and G2/M checkpoint control. These findings support our earlier co-expression analyses, reinforcing the role of *EXT1* in safeguarding replication fork stability and coordinating DNA repair, particularly within stromal-like tumor niches. Such enrichment is consistent with the observation that *EXT1*-high gliomas exhibit resistance to genotoxic agents, likely through enhanced tolerance to replication stress (Fig. 8A-C). In contrast, *EXT2* showed strong enrichment in cytoskeleton- and motility-related pathways, including EMT, apical junction remodeling, and Rho GTPase-driven actin reorganization. Additional enrichment in complement and inflammatory response pathways further suggests that EXT2 contributes to a pro-invasive and immune-modulatory tumor phenotype (Fig. 8D-F).

### 3.6 *EXT1* and *EXT2* Co-expression Modules Define Distinct Functional Programs

**Figure 9:**
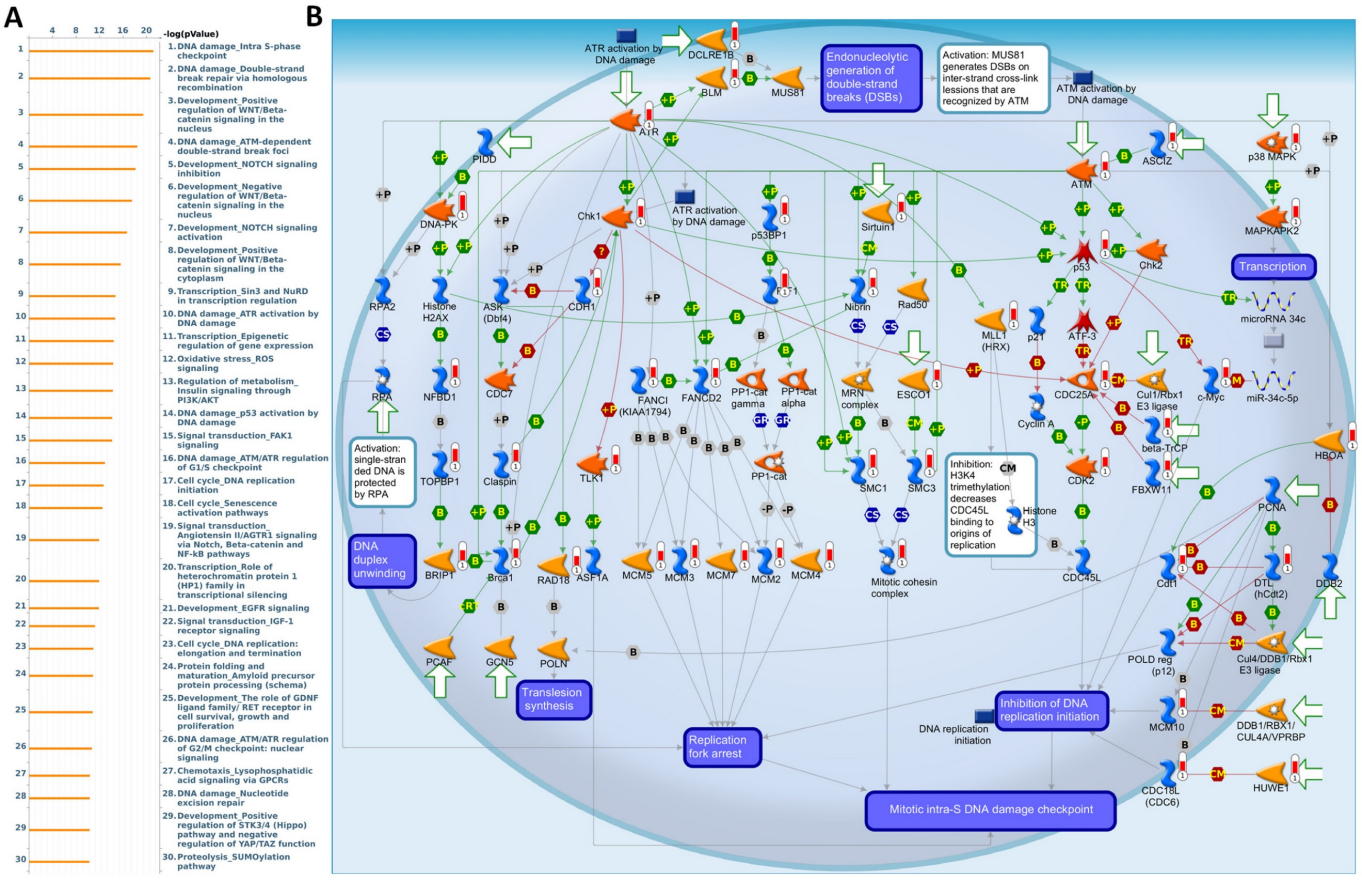
** MetaCore pathway enrichment analysis of the *EXT1* co-expression genes in gliomas patients from TCGA.** (A) Top 30 enriched pathways identified by MetaCore using genes co-expressed with *EXT1* ranked by -log₁₀(p value). (B) Representative MetaCore process network map highlighting the “DNA damage - intra S-phase checkpoint” pathway.

**Figure 10:**
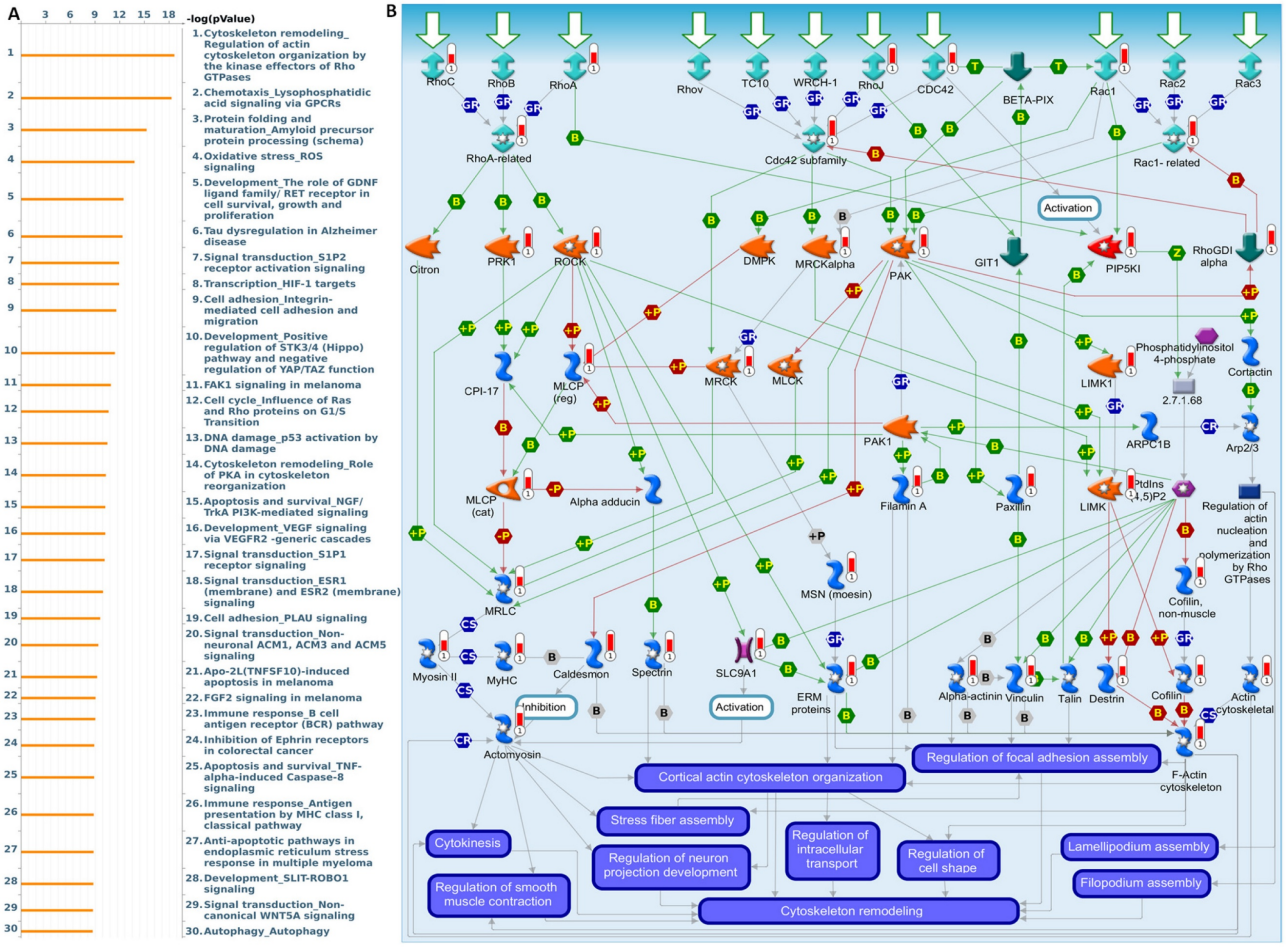
** MetaCore pathway enrichment analysis of the *EXT2* co-expression genes in gliomas patients from TCGA.** (A) Top 30 enriched pathways identified by MetaCore using genes co-expressed with *EXT2* ranked by -log₁₀(p value). (B) Representative MetaCore process network map highlighting the “Cytoskeleton remodeling - Regulation of actin cytoskeleton by Rho GTPases” pathway.

To further validate and investigate the functional divergence between *EXT1* and *EXT2*, we performed genome-wide co-expression analyses using TCGA-Glioma transcriptomic data. Genes exhibiting strong positive correlations with *EXT1* or* EXT2* (|R| > 0.4, p < 0.001) were independently subjected to pathway enrichment analysis using the MetaCore platform. EXT1-associated co-expression networks were highly enriched for pathways related to DNA replication initiation, replication fork arrest, and checkpoint activation particularly involving the ATR-CHK1 axis (Fig. 9A, B). Notable co-regulated genes included members of the MCM2-7 (mini-chromosome maintenance 2-7) helicase complex, *CDC45* (cell division cycle 45), *BRIP1* (BRCA1-interacting protein 1), *FANCI* (Fanconi anemia, complementation group 1), and *GINS* (GINS complex subunit) components, which collectively form the core DNA replication machinery essential for origin licensing and fork stabilization. MetaCore pathway mapping highlighted *EXT1* as part of a broader regulatory module controlling intra-S phase checkpoint activation, homologous recombination repair, and mitotic spindle assembly, suggesting that *EXT1* may confer resistance to replication stress through coordination of checkpoint fidelity and damage tolerance. Pathway enrichment terms such as “DNA duplex unwinding”, “Double-strand break repair,” “G2/M checkpoint,” and “Translesion synthesis” further support a role for *EXT1* in maintaining genomic integrity under replication perturbation. In contrast, EXT2-associated gene modules were significantly enriched in cytoskeletal remodeling pathways, including Rho GTPase, *PAK1* (p21-activated kinase 1), and *LIMK1* (LIM domain kinase 1) signaling cascades (Fig. 10A, B). Co-expressed genes such as *ROCK1* (Rho-associated coiled-coil kinase 1), *RAC1* (Ras-related C3 botulinum toxin substrate 1), *ACTN4* (alpha-actinin-4), and *ARP2/3* (actin-related protein 2/3) complex members are involved in actin polymerization, lamellipodium extension, and focal adhesion dynamics, hallmarks of invasive and mesenchymal-like glioma phenotypes. Pathway enrichment terms including “Actin cytoskeleton organization”, “Stress fiber assembly”, and “Cell-substrate adhesion” were strongly overrepresented, reinforcing *EXT2*'s role in tumor cell migration, plasticity, and ECM interactions. The detailed MetaCore pathway maps for *EXT1*-related modules are presented in Supplementary Figures S4, illustrating enriched signaling circuits involved in replication fork stabilization, *ATR-CHK1* activation, and mitotic checkpoint control. Pathway maps for *EXT2* are shown in Supplementary Figures S5, highlighting networks regulating Rho GTPase-mediated actin remodeling, cell-substrate adhesion, and motility signaling. The specific correlated genes contributing to each pathway, along with corresponding enrichment scores and adjusted p values, are listed in Supplementary Table S1-2, providing a quantitative framework that links individual *EXT1/2* interactors to their respective functional modules.

### 3.7 Drug Sensitivity and Therapeutic Implications of *EXT1/2* Expressions

**Figure 11:**
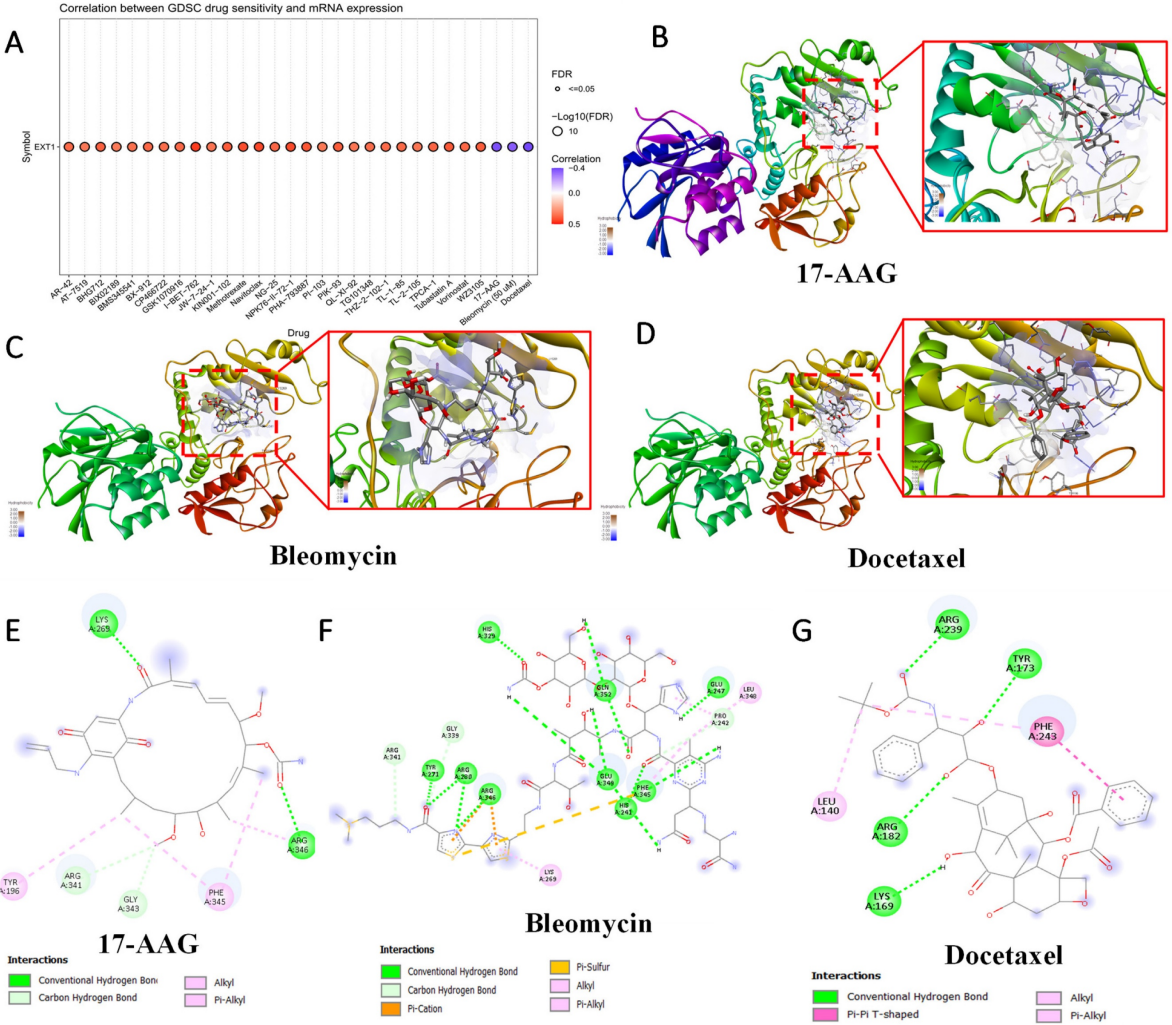
** Molecular docking analysis of selected compounds with the *EXT1* protein.** (A) Drug Sensitivity (GDSC) analysis showing correlations between *EXT1* mRNA expression and predicted IC₅₀ responses across pharmacologic agents. (B-C) 3D docking poses of 17-AAG, bleomycin, and pentostatin, respectively, showing the binding orientation within the *EXT1* active site. The protein secondary structure is color-coded, with magnified insets highlighting ligand placement in the binding pocket. (D) 2D interaction diagrams for 17-AAG (E), bleomycin (F), and docetaxel (G) depicting key molecular interactions, including conventional hydrogen bonds (green), carbon hydrogen bonds (light blue), alkyl and π-alkyl interactions (pink), π-sulfur interactions (yellow), and π-π T-shaped stacking (purple). Residues involved in ligand binding are labeled, with corresponding interaction types indicated in the legend.

**Figure 12:**
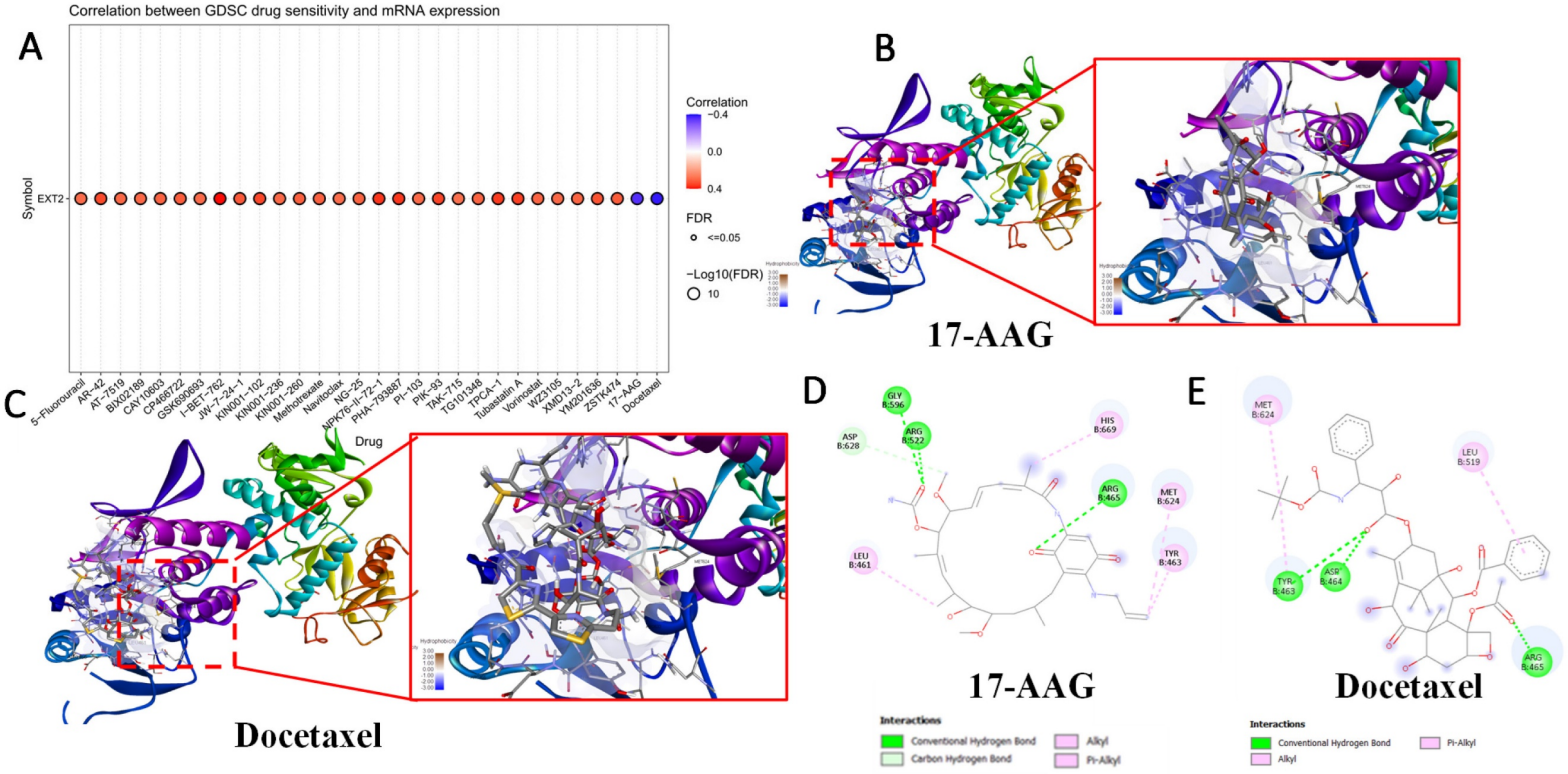
** Molecular docking analysis of selected compounds with the *EXT2* protein.** (A) Drug Sensitivity (GDSC) analysis showing correlations between *EXT2* mRNA expression and predicted IC₅₀ responses across pharmacologic agents. (B-C) 3D docking poses of 17-AAG, docetaxel, and their overlay, respectively, showing the binding orientation within the active site. The protein secondary structure is color-coded, with magnified insets highlighting ligand placement in the binding pocket. (D, E) 2D interaction diagrams for 17-AAG and docetaxel, depicting key molecular interactions, including conventional hydrogen bonds (green), alkyl interactions (pink), and π-alkyl interactions (purple). Residues involved in ligand binding are labeled, with corresponding interaction types as indicated in the legend.

To evaluate the clinical relevance of *EXT1* and *EXT2* in the context of therapeutic responses, we analyzed drug sensitivity correlations using the Genomics of Drug Sensitivity in Cancer (GDSC) database. Pearson correlation analysis between *EXT1/2* expressions and IC_50_ values across 265 compounds revealed distinct pharmacogenomic profiles for each gene. *EXT1* expression was broadly and positively correlated with resistance to several DNA-damaging agents and antimitotic compounds, including bleomycin, etoposide, and docetaxel (Fig. 11). To explore potential drug-target interactions, we performed molecular docking simulations for *EXT2* with selected compounds showing strong GDSC correlations (Fig. 12). Docetaxel demonstrated the highest predicted binding affinity to *EXT2* (-8.4 kcal/mol), supported by extensive hydrophobic contacts, suggesting a stable, albeit indirect, interaction with *EXT2*-associated molecular surfaces. 17-AAG and bleomycin exhibited moderate binding (-7.1 and -7.0 kcal/mol, respectively), with bleomycin forming the most significant number of hydrogen bonds but showing the weakest overall affinity, indicating that binding stability is not solely determined by the interaction quantity but by structural complementarity and energy minimization. Importantly, neither *EXT1* nor *EXT2* expression showed a significant correlation with temozolomide (TMZ) sensitivity, implying that their resistance mechanisms operate independently of the canonical MGMT-mediated alkylator response axis. This observation raises the possibility that *EXT1/2* expressions could serve as biomarkers for patient stratification in alternative treatment contexts, especially where resistance to TMZ is observed despite the MGMT methylation status. These pharmacogenomic and structural modeling findings reinforce the distinct therapeutic implications of *EXT1* and* EXT2*. *EXT1*-high tumors may be vulnerable to synthetic lethality approaches involving checkpoint inhibition (*CHK1* or *ATR* inhibitors), whereas *EXT2*-driven gliomas might respond to combinatorial regimens targeting cytoskeletal remodeling or focal adhesion dynamics 73-75. These insights support the potential of *EXT1* and *EXT2* as predictive biomarkers and targets for rational drug design in gliomas.

### 3.8 IHC Validation of *EXT1* Expression in Glioma Progression

**Figure 13:**
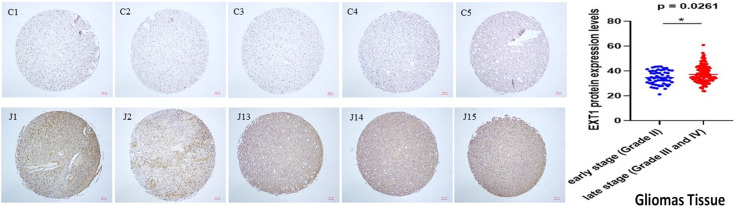
** IHC staining of the *EXT1* protein across gliomas tissue microarrays.** Left panel: Representative *EXT1* staining in early-stage (WHO grade II; C1-C5) and late-stage (WHO grades III-IV; J1-J5) glioma cores from a tissue microarray. Early-stage tumors show relatively weaker *EXT1* immunoreactivity, whereas late-stage tumors display stronger and more diffuse cytoplasmic staining. Right panel: Quantitative comparison of *EXT1* protein expression scores between early-stage (blue dots) and late-stage (red dots) gliomas, demonstrating significantly higher *EXT1* levels in advanced tumors (p = 0.0261).

While both *EXT1* and *EXT2* were found to be upregulated in gliomas, we prioritized *EXT1* for immunohistochemical (IHC) validation due to its stronger transcriptomic association with immune suppression, stromal activation, and clinical outcomes. *EXT1* also exhibited more robust staining in pilot assays using validated antibodies. Therefore, we performed an IHC analysis on a TMA containing glioma samples spanning low- to high-grade tumors: WHO grade II (astrocytomas), grade III (anaplastic astrocytomas), and grade IV (glioblastomas). Representative IHC images (Fig. 13) showed minimal *EXT1* expression in low-grade gliomas, whereas high-grade tumors exhibited strong cytoplasmic staining, predominantly localized to non-neuronal stromal regions rather than neuronal compartments. A quantitative evaluation confirmed a statistically significant increase in *EXT1* protein levels in high-grade tumors compared to low-grade tumors (p = 0.0261). Nonetheless, the complementary roles of *EXT2* in ECM remodeling and angiogenesis merit further investigation at the protein level.

## 4. Discussion

Despite significant advances in molecular and epigenomic profiling, the glycosylation landscape of gliomas remains largely uncharacterized 76. Most prior studies focused on extracellular HS modifiers such as heparinase or *SULF2* 77, 78, which remodel the tumor matrix and influence angiogenesis and invasion 79, 80. However, the core polymerizing enzymes of the HS biosynthetic pathway, *EXT1* and *EXT2*, have been widely overlooked. By integrating bulk cohorts, single-cell atlases, methylome and network analyses, pharmacogenomics, limited structure-guided modeling, and targeted protein readouts, we delineate a nonredundant dual-axis organization of *EXT1* and *EXT2.* Both enzymes are elevated and associated with inferior survival. Single-cell maps and histology converge on *EXT1* enrichment in endothelial, pericytic, and stromal territories and glia, whereas *EXT2* concentrates in malignant mesenchymal-like states that co-express contractile and adhesion machinery. This partition provides a substrate for immune exclusion, angiogenic and matrix conditioning on one side, and traction generation and motility on the other, which rationalizes the observed outcome associations.

Pathway and network analyses revealed that *EXT1* program aligns with E2F and G2M cell-cycle modules, replication-stress tolerance, and checkpoint biology, consistent with dependence on ATR-CHK1 under genotoxic pressure. The *EXT2* program aligns with PI3K-AKT-MTOR signaling, focal adhesion, Rho-directed actin remodeling, and oxidative metabolism, consistent with adhesion competence, cytoskeletal plasticity, and invasive fitness. Meanwhile, both enzymes sit within conserved HS biosynthetic machinery, yet interactome neighborhoods and epigenetic features diverge. *EXT1* shows promoter and gene-body hypomethylation correlated with transcriptional upregulation in aggressive disease, while *EXT2* clusters with glypican and syndecan modules that scaffold adhesion complexes. These observations argue that upstream HS polymerization is not a uniform Golgi-confined process but a context-dependent regulator that biases downstream signaling along stromal and mesenchymal axes. These IHC results thus provide wet-lab validation of our computational model, confirming that *EXT1* activation is not merely transcriptional but extends to the protein level within malignant glioma compartments. Our pharmacogenomic analyses provide translational insights into the therapeutic relevance of this model. *EXT1*-high tumors displayed resistance to DNA-damaging agents such as bleomycin and etoposide and mitotic inhibitors like docetaxel, supporting a replication stress-tolerant phenotype 81-83. Conversely, *EXT2*-high tumors exhibited resistance to microtubule-disrupting drugs such as vinblastine and paclitaxel, consistent with cytoskeletal resilience 84. These patterns not only highlight *EXT1* and *EXT2* as predictive biomarkers of drug responses but also suggest that targeting replication stress checkpoints (ATR/CHK1 inhibitors) or actin-myosin pathways (ROCK/FAK inhibitors) may selectively sensitize *EXT1*- or *EXT2*-driven gliomas, respectively 85. Such stratification could guide personalized therapy in clinical settings where current genomic markers (IDH and MGMT) offer limited predictive resolution.

The novelty of this study lies in revealing that *EXT1* and *EXT2*, traditionally viewed as redundant HS polymerases, are in fact spatially partitioned regulators of glioma aggressiveness. Through integrated multi-omics and single-cell approaches, we demonstrated that glycosylation interfaces directly with DNA replication, immune modulation, and cytoskeletal dynamic dimensions previously disconnected in glioma biology. The identification of an *EXT1* or *EXT2* axis glycosylation circuit introduces a new conceptual framework linking ECM synthesis to intracellular oncogenic signaling. Importantly, this study establishes a foundation for glycan-centered therapeutic targeting, positioning the EXT family as a previously unrecognized vulnerability in cancer pathogenesis 86, 87. Several limitations define the next-phase agenda. Reliance on public cohorts carries batch and composition biases despite cross-cohort validation. Transcriptomes are imperfect surrogates for glycan fine structure, since chain length, sulfation pattern, and site occupancy are post-transcriptionally controlled. Spatial resolution at vascular-stromal interfaces remains limited in available single-cell datasets. Pharmacogenomic and docking signals are correlative and structural in nature, requiring genetic perturbation and rescue experiments for confirmation.

In conclusion, this study integrates bulk cohorts, single-cell topologies, pathway and network models, and immunohistochemistry to provide a coherent biological narrative explaining adverse survival associations and linking glycosylation to immune exclusion, angiogenesis, and invasion. The framework yields tractable axis-specific strategies: in *EXT1*-dominant contexts, testing combinations that impair fork protection and checkpoint fidelity, such as *ATR* or *CHK1* inhibitors layered on genotoxic backbones; in *EXT2*-dominant contexts, targeting focal adhesion assembly, Rho-actomyosin effectors, or allied cytoskeletal circuits to blunt invasive fitness. By aligning multi-omic signals with niche topology, the *EXT1* or *EXT2* axis model offers a rigorous template for glyco-oncology in glioma and a roadmap for prospective validation in organoids, patient-derived xenografts, and early translational research.

## Supplementary Material

Supplementary figures and table.

## Figures and Tables

**Figure 14:**
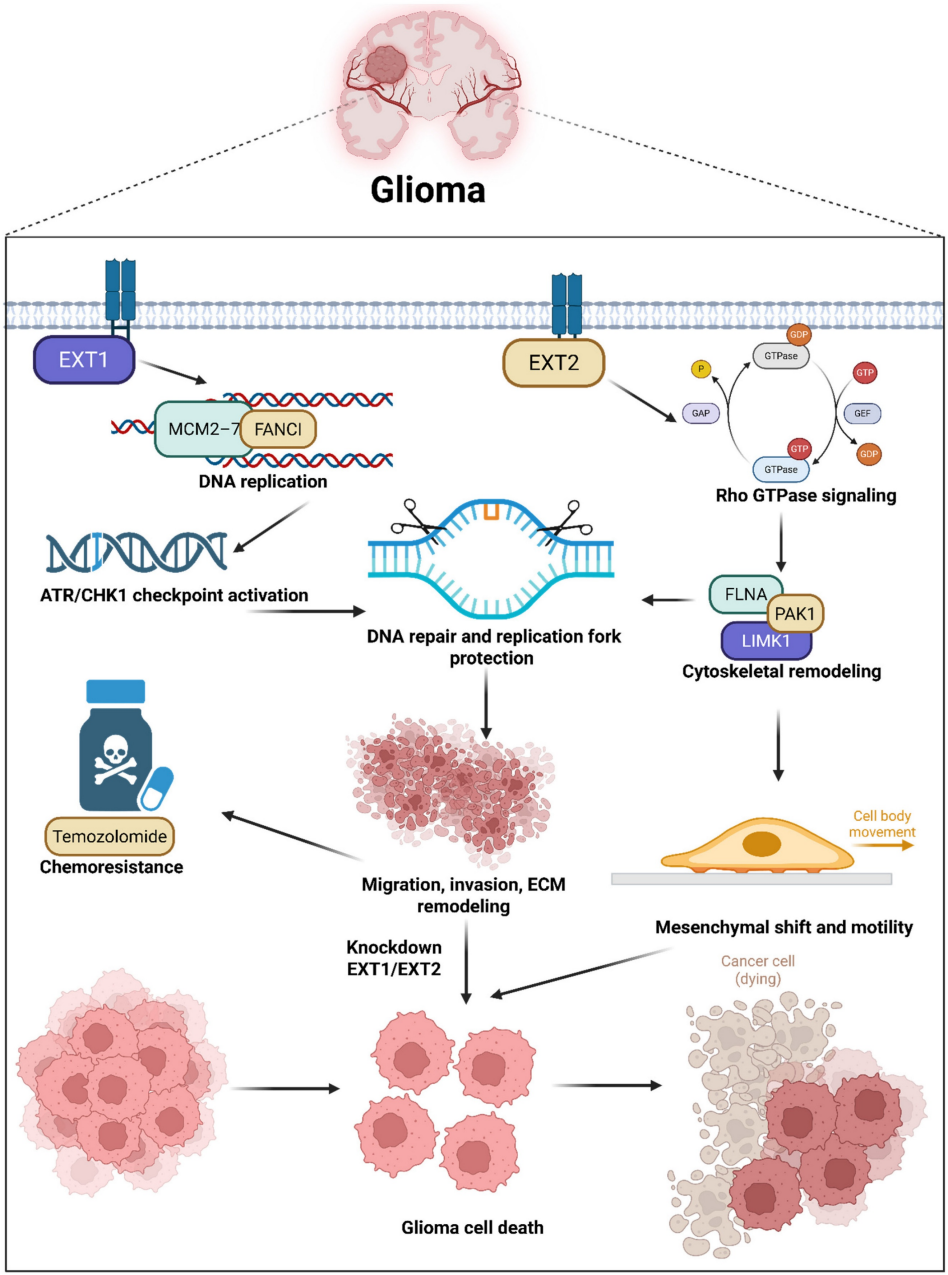
** Schematic illustration summarizing the proposed dual-axis model of *EXT1*'s and *EXT2*'s functions in glioma biology.** *EXT1* and *EXT2* exhibit distinct yet complementary oncogenic roles within the glioma ecosystem. These parallel programs orchestrate ECM remodeling, invasion, and tumor progression, with *EXT1* sustaining replication-stress adaptations and stromal stability, while *EXT2* drives the mesenchymal transition and invasiveness. This figure illustrates how the *EXT1-EXT2* glycosylation axis integrates DNA damage tolerance and cytoskeletal dynamics, shaping glioma heterogeneity and therapeutic resistance.

## Abbreviations

ANOVA: analysis of variance
ATR: ataxia telangiectasia and Rad3-related protein
CGGA: Chinese Gliomas Genome Atlas
CHK1: checkpoint kinase 1
CIBERSORT: Cell-type Identification By Estimating Relative Subsets Of RNA Transcripts
CpG: cytosine-phosphate-guanine
CTLA4: cytotoxic T-lymphocyte-associated protein 4
CTRP: Cancer Therapeutics Response Portal
DNA-seq: DNA sequencing
DSS: disease-specific survival
ECM: extracellular matrix
EXT1: exostosin-1
EXT2: exostosin-2
ESTIMATE: Estimation of STromal and Immune cells in MAlignant Tumors using Expression data
FAK: focal adhesion kinase
FFPE: formalin-fixed paraffin-embedded
FDR: false discovery rate
GBM: glioblastoma multiforme
GDSC: Genomics of Drug Sensitivity in Cancer
GO: gene ontology
GTEx: Genotype-Tissue Expression
H-score: histological score
HR: hazard ratio
HS: heparan sulfate
IHC: immunohistochemistry
IDH: isocitrate dehydrogenase
KEGG: Kyoto Encyclopedia of Genes and Genomes
LASSO: Least Absolute Shrinkage and Selection Operator
LGG: low-grade glioma
MD: molecular dynamics
MM-PBSA: Molecular Mechanics Poisson-Boltzmann Surface Area
OPC: oligodendrocyte precursor cell
OS: overall survival
PCA: principal component analysis
PD-1: programmed cell death protein 1
PD-L1: programmed death-ligand 1
PFI: progression-free interval
RNA-seq: RNA sequencing
Rho: Ras homolog family of GTPases
RMSD: root mean square deviation
SASA: solvent-accessible surface area
TCGA: The Cancer Genome Atlas
TGF-β: transforming growth factor beta
TIGIT: T cell immunoreceptor with Ig and ITIM domains
TIMER: Tumor Immune Estimation Resource
TMA: tissue microarray
TMZ: temozolomide
TPM: transcripts per million
TME: tumor microenvironment
UMAP: Uniform Manifold Approximation and Projection
WHO: World Health Organization
