# Supplementary material for: Multi-Omics and Single-Cell Dissection of Exostosin Glycosyltransferases (EXT1/EXT2) Reveals Divergent Oncogenic Roles and Therapeutic Vulnerabilities in Gliomas
Source: J Cancer. 2026 Jan 1;17(1):177–96. doi: 10.7150/jca.123965 (PMC12719590; doi:10.7150/jca.123965)
Supplement: Supplementary file 1 — Supplementary figures and table. [file jcav17p0177s1.pdf]

Supplementary Data

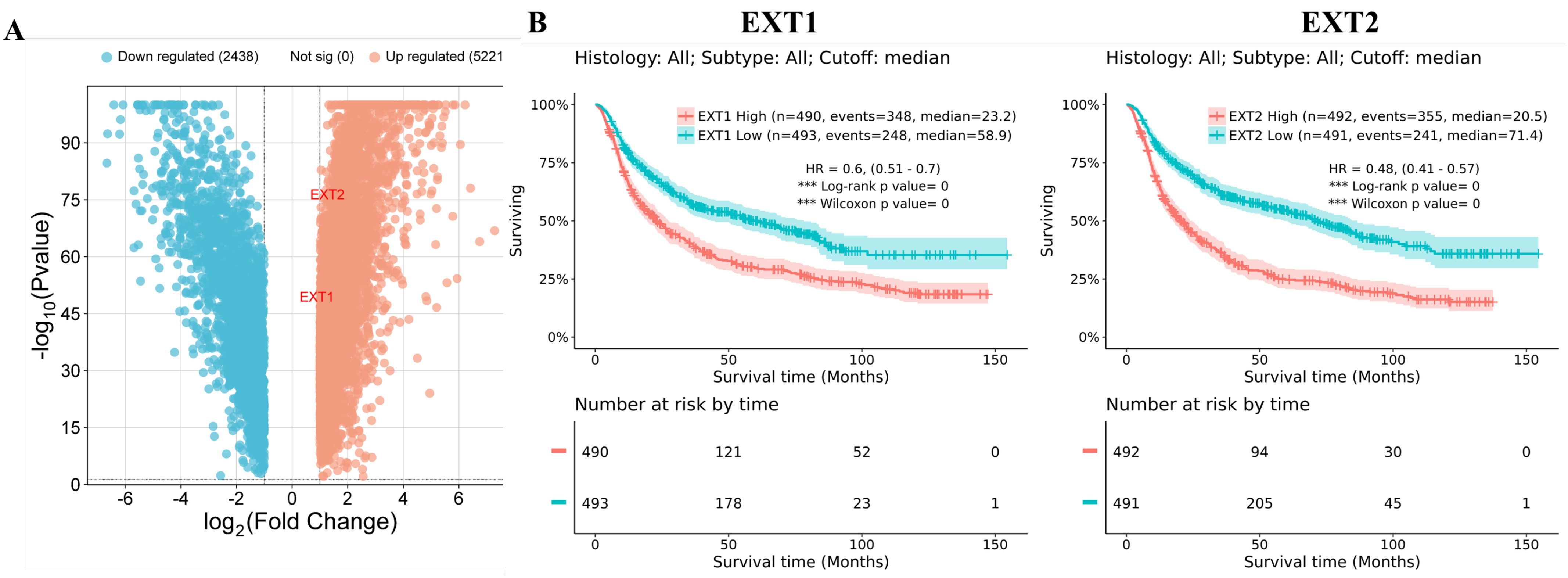

Supplementary Figure S1: **Differential expressions and prognostic significance of *EXT1* and *EXT2* in gliomas.** (A) Volcano plot showing differential gene expressions between glioma and normal brain tissues based on TCGA transcriptomic data. *EXT1* and *EXT2* are significantly upregulated (log2FC > 1.5, adjusted p < 0.001), highlighting their potential oncogenic relevance. (B) Kaplan-Meier survival analysis of overall survival (OS) in glioma patients stratified by *EXT1* expression (TCGA cohort, median cutoff). Patients with high EXT1 expression (n = 490, median OS = 23.2 months, 348 events) exhibited significantly poorer survival compared to the low-expression group (n = 493, median OS = 58.9 months, 248 events). The hazard ratio (HR = 0.60, 95% CI = 0.51–0.70; log-rank p < 0.001) indicates a ~40% higher mortality risk in EXT1-high cases. Kaplan-Meier analysis of *EXT2* expression using the same median cutoff approach. EXT2-high tumors (n = 492, median OS = 20.5 months, 355 events) demonstrated substantially reduced survival relative to *EXT2*-low tumors (n = 491, median OS = 71.4 months, 241 events; HR = 0.48, 95% CI = 0.41–0.57; log-rank p < 0.001). Shaded areas denote 95% confidence intervals.

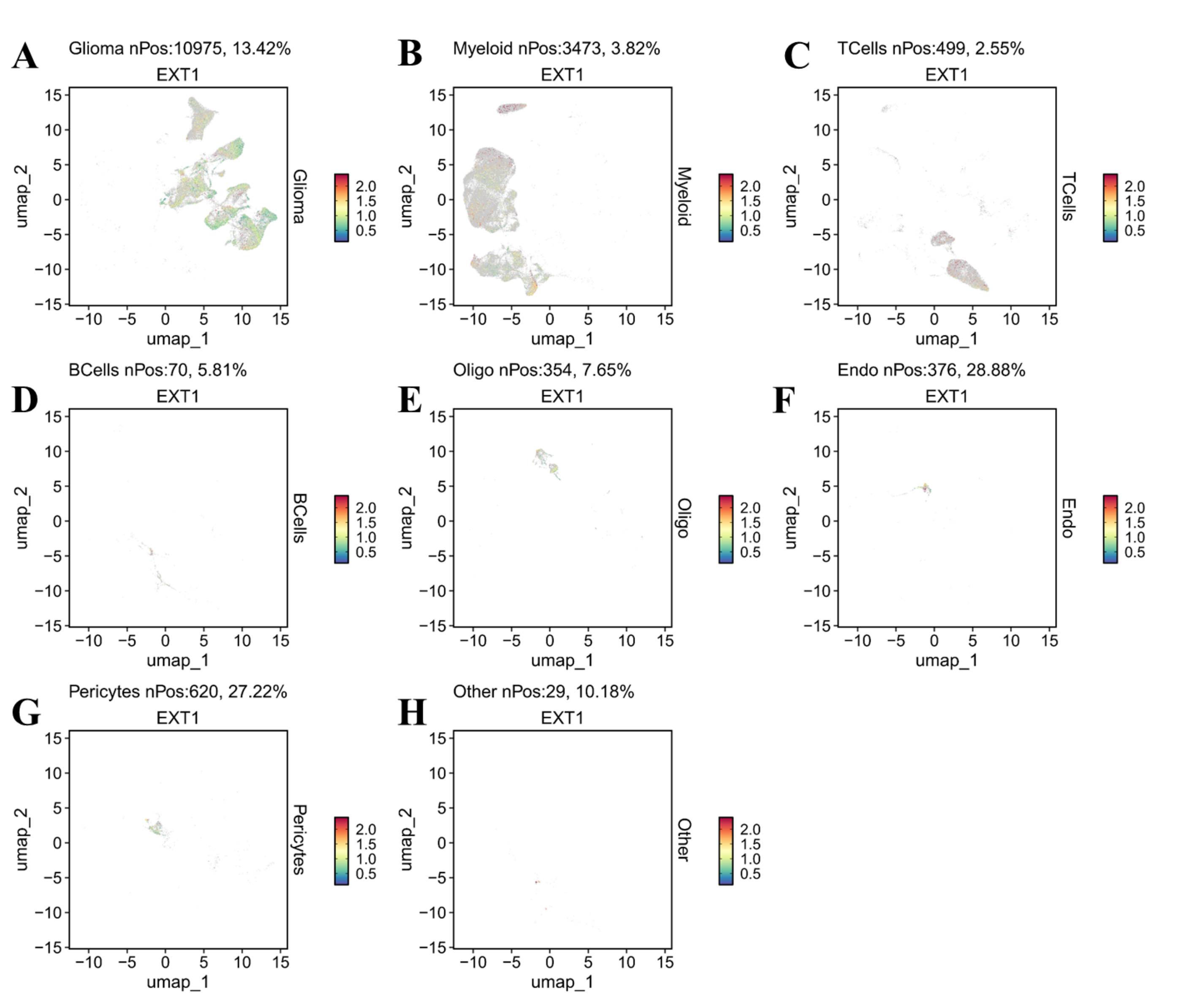

Supplementary Figure S2: **Single-cell expression patterns of *EXT1* in across cell types.** (A–H) UMAP feature plots showing EXT1 expression across major cellular compartments, including malignant glioma cells (A), myeloid cells (B), T cells (C), B cells (D), oligodendrocytes (E), endothelial cells (F), pericytes (G), and other minor populations (H). Each dot represents a single cell colored by normalized *EXT1* transcript level (blue = low, red = high). EXT1 expression is markedly enriched in endothelial and pericyte clusters, indicating a vascular–stromal distribution consistent with a role in heparan-sulfate–mediated extracellular matrix organization, angiogenic remodeling, and immune-exclusion signaling..

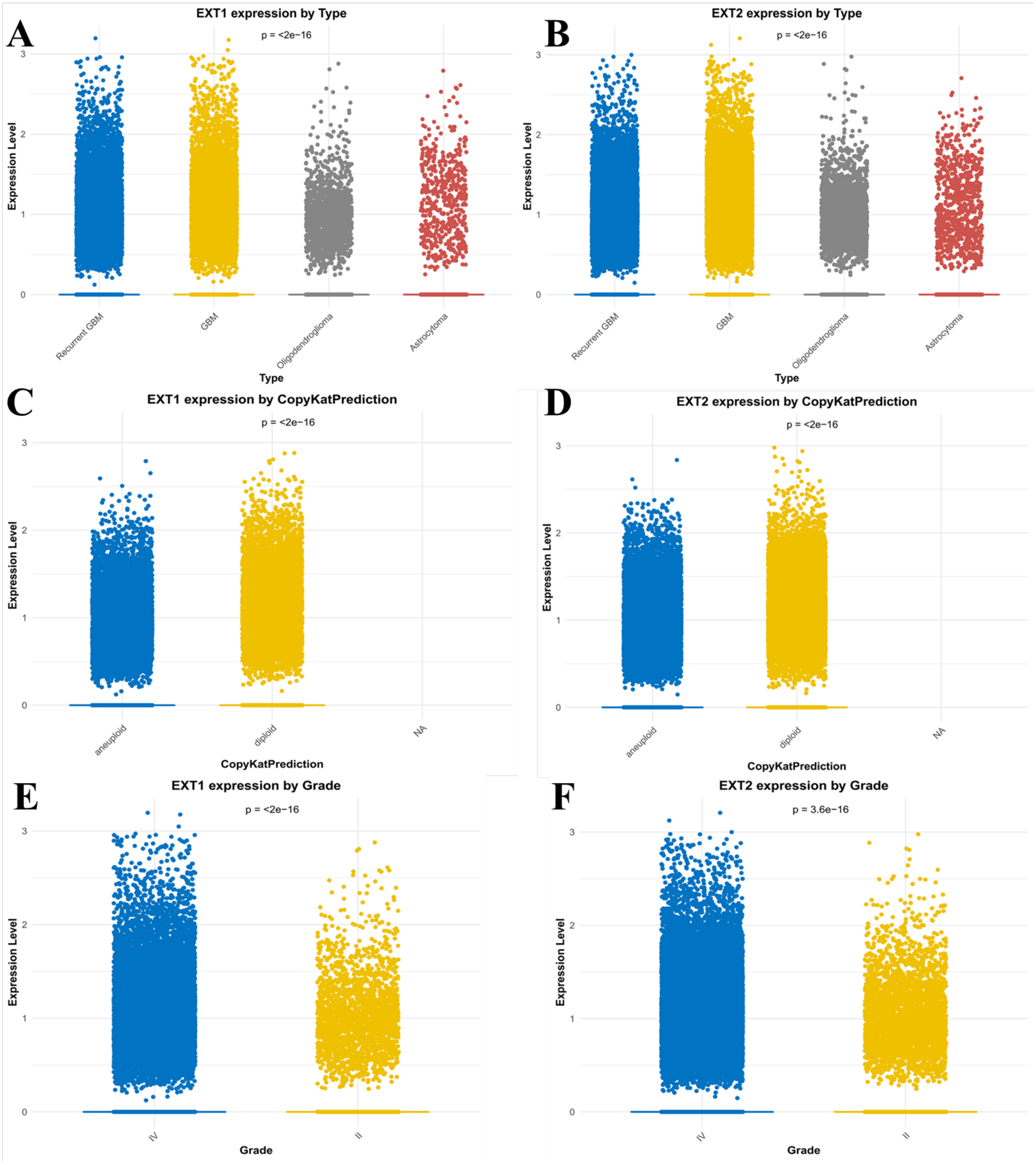

Supplementary Figure S3: **Comparition of *EXT1* and *EXT2* expressions by tumor type, genomic status, and grade.** (A, B) Scatterplots displaying *EXT1* (A) and *EXT2* (B) expressions among recurrent GBM, primary GBM, oligodendroglioma, and astrocytoma samples. Expressions of *EXT1* (C) and *EXT2* (D) stratified by the CopyKat-predicted genomic status (diploid vs. aneuploid). Expressions of *EXT1* (E) and *EXT2* (F) according to WHO tumor grade. Both genes show significantly elevated expression in recurrent and high-grade gliomas ($p < 2 \times 10^{-16}$). *EXT1* is associated with stromal and endothelial aneuploid clones, while *EXT2* is correlated with copy-gain mesenchymal tumor populations, supporting their cooperative roles in vascular stabilization and tumor invasiveness.

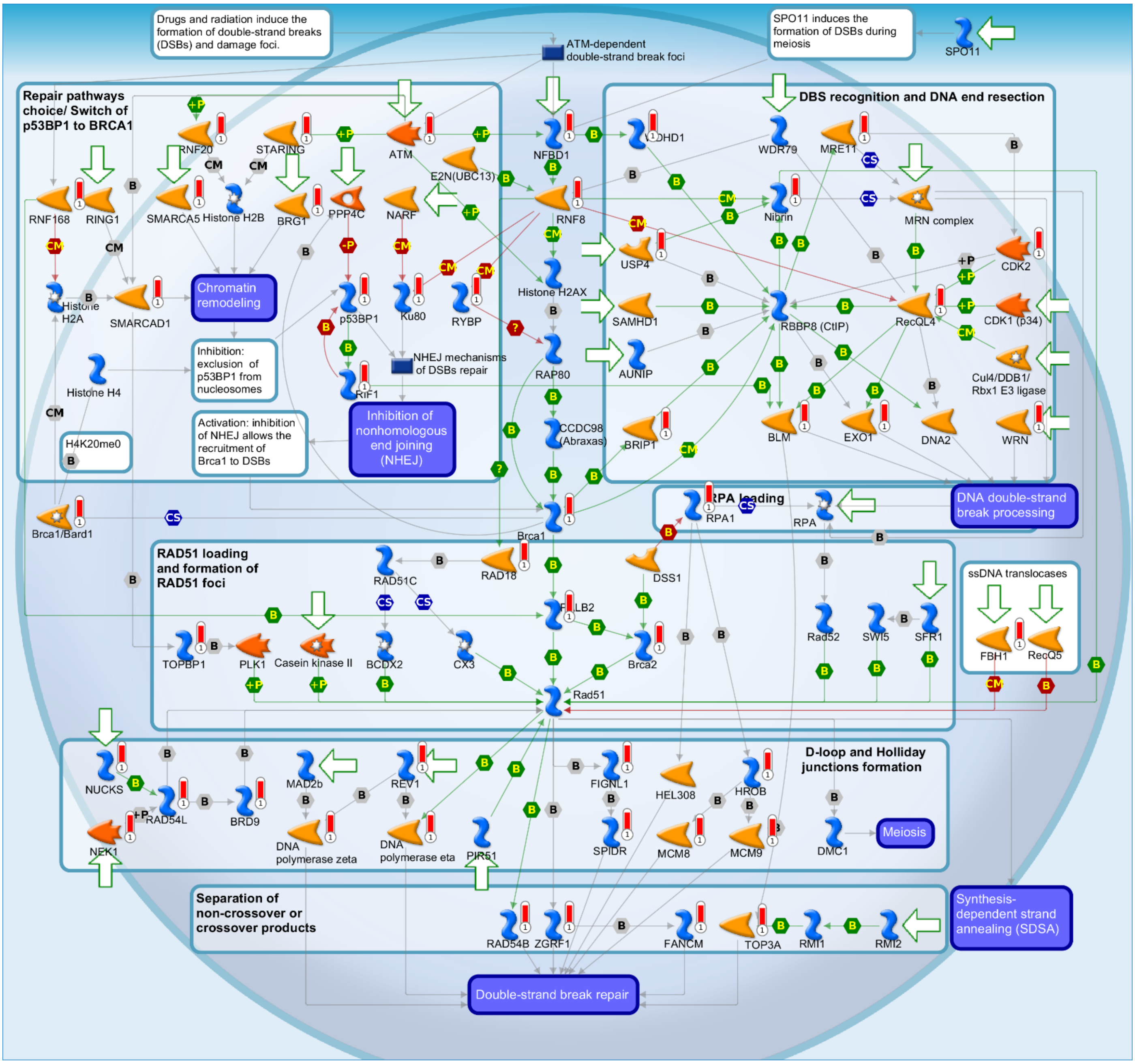

Supplementary Figure S4. MetaCore pathway enrichment analysis of the EXT1 co-expression genes in glioma patients from TCGA. (A) Top 25 enriched pathways identified by MetaCore using genes co-expressed with EXT1 ranked by –log$_{10}$(p value). (B) Representative MetaCore process network map highlighting the “DNA damage_Double-strand break repair via homologous recombination” pathway.

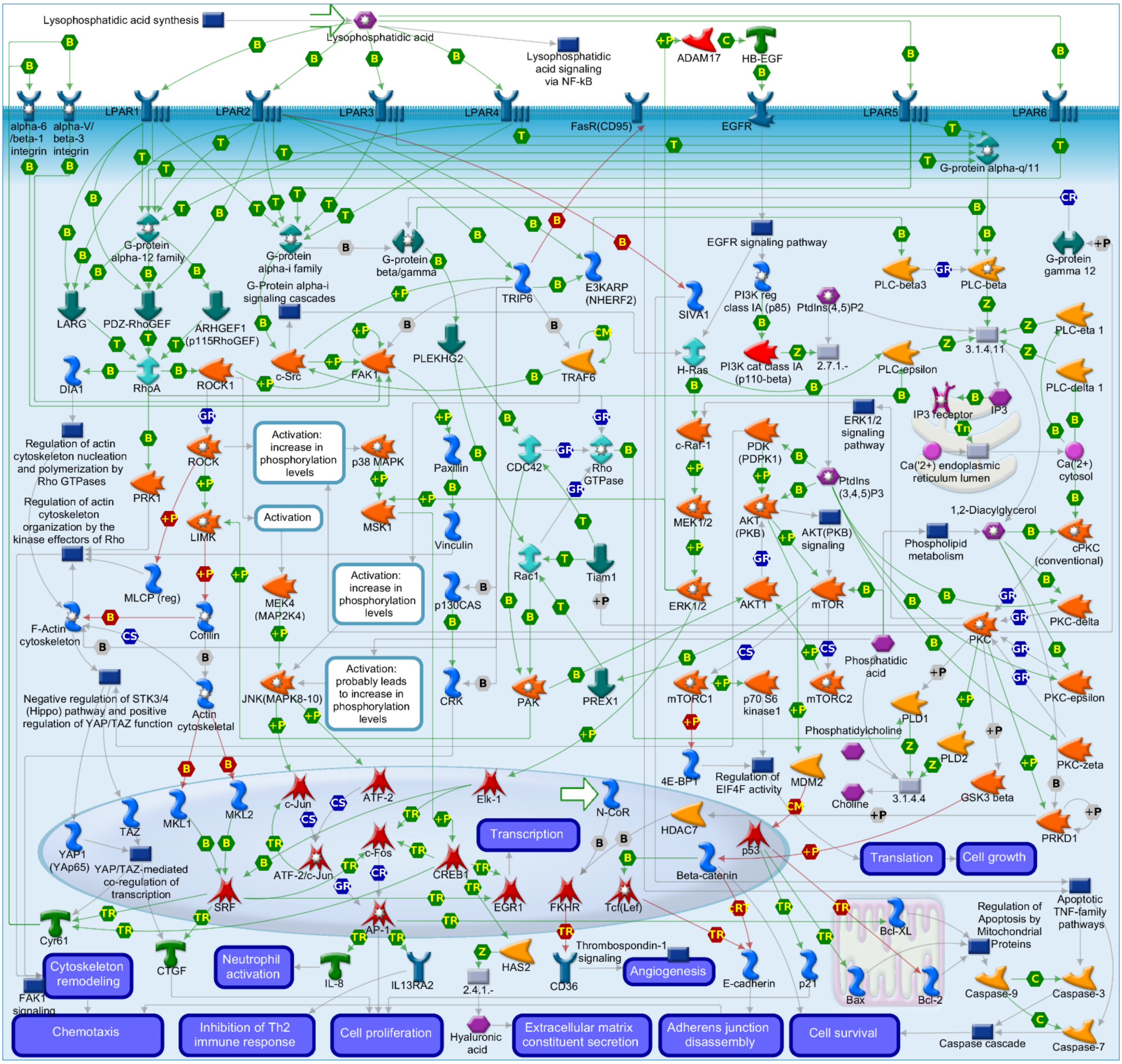

Supplementary Figure S5. MetaCore pathway enrichment analysis of the EXT2 co-expression genes in glioma patients from TCGA. (A) Top 25 enriched pathways identified by MetaCore using genes co-expressed with EXT1 ranked by $-\log_{10}$(p value). (B) Representative MetaCore process network map highlighting the “Chemotaxis_Lysophosphatidic acid signaling via GPCRs” pathway.

Supplementary Table S1: Pathway analysis of genes co-expressed with *EXT1* in gliomas in TCGA databases using the MetaCore database (with a p value of < 0.05 set as the cutoff value).

#	Maps	pValue	Network Objects from Active Data
**1**	DNA damage_Intra S-phase checkpoint	7.16E-22	MCM3, RAD18, RIF1, NFBD1, Brca1, MCM5, SMC1, BLM, DTL (hCdt2), MCM4, HUWE1, MCM10, beta-TrCP, DNA-PK, CDH1, DCLRE1B, CDC25A, ATR, TOPBP1, p53BP1, CDK2, CDC18L (CDC6), HBOA, MLL1 (HRX), Chk1, Mitotic cohesin complex, Sirtuin1, MAPKAPK2, MCM7, BRIP1, SMC3, FANCD2, FANCI (KIAA1794), ATM, ASCIZ, ESCO1, MCM2, Cdt1, p53, c-Myc, Claspin, Nibrin, TLK1, p38 MAPK, FBXW11
**2**	DNA damage_Double-strand break repair via homologous recombination	2.44E-21	RAD18, ZGRF1, RIF1, DNA polymerase zeta, NFBD1, SPIDR, Brca1, Brca2, BLM, STARING, PALB2, SMARCAD1, MCM8, RNF168, BRG1, FBH1, REV1, EXO1, TOPBP1, DNA polymerase eta, NUCKS, WRN, p53BP1, CDK2, FIGNL1, WDHD1, Ku80, MRE11, TOP3A, BRIP1, RYBP, RAD54B, RAD54L, BRD9, ATM, RNF8, NEK1, RecQL4, MCM9, SMARCA5, Brca1/Bard1, RPA1, FANCM, Nibrin, RNF20, HROB, USP4
**3**	Development_Positive regulation of WNT/Beta-catenin signaling in the nucleus	3.99E-20	Casein kinase II, alpha chains, SOX11, TBL1X, CBP, LRP5/LRP6, TWA1, Tcf(Lef), PIAS4, XIAP, beta-TrCP, Alpha-1 catenin, TCF7L2 (TCF4), FOXM1, Jade-1, BCL9/B9L, VCP, FOXK1, TLE, ZIP-kinase, p300, CBP/P300, NCOA2 (GRIP1/TIF2), DLL4, Dsh, Casein kinase I alpha, UBR5, Sirtuin1, GSK3 beta, HDAC2, UCHL5, FOXK2, FOXO3A, TBLR1, SOX4, HDAC1, APPL, ERK2 (MAPK1), LRRFIP2, SOX9, Frizzled, JRK
**4**	DNA damage_ATM-dependent double-strand break foci	4.35E-19	JMJD2A, CBP, NPL4, NFBD1, Brca1, PARP-1, BRM, STARING, PIAS4, RNF168, BRG1, KDM2A, BAF180, VCP, PIAS1, ENL, WHSC1, p300, DOT1, p53BP1, TIF1-beta, HBXAP, USP7, ATM, RNF8, Mi-2 alpha, RING2, SET8, SMARCA5, Brca1/Bard1, Bcl-10, BAZ1A, HERC2, BRCC36, p53, BAT3, Bard1, Nibrin, RNF20
**5**	Development_NOTCH signaling inhibition	8.8E-19	Casein kinase II, alpha chains, NOTCH1 (NICD), AKT1, NOTCH1 receptor, c-Cbl, BACE1, FBXW7, SMRT, Presenilin 2, MAML1, 14-3-3 zeta/delta, DYRK1a, J1ICD, HDAC4, LSD1, NOTCH2 receptor, DLL4, L3MBTL3, RBB2, NOTCH2 (2ICD), KCTD10, NOTCH3 (3ICD), Sirtuin1, GSK3 beta, HDAC2, TACC3, Itch, MMP-14, NOTCH1 precursor, N-CoR, Skp2/TrCP/FBXW, WDR48, USP12, HDAC1,

			AKT(PKB), Cullin 3, RBP-J kappa (CBF1), p53, Jagged1, c-Src, CtBP1, DVL-2, SHARP (SPEN), Nibrin, p38 MAPK, APP-C59 (AICD), NUMB, NOTCH3 receptor
**6**	Development_Negative regulation of WNT/Beta-catenin signaling in the nucleus	3.66E-18	ZNF703, KDM2, TBL1X, LRP5/LRP6, Casein kinase I delta, AKT1, TRRAP, c-Cbl, BACH1, Tcf(Lef), Alpha-1 catenin, TCF7L2 (TCF4), 14-3-3, Jade-1, NF-AT3(NFATC4), TRIM33, BCL9/B9L, GLI-3R, PC1-CTT, CHD8, TLE, SENP2, CBP/P300, Kaiso, Dsh, Menin, NF-AT5, GSK3 beta, HDAC2, SOX2, FOXO3A, HIC1, LATS2, SENP1, RANBP3, CtBP, TBLR1, HDAC1, Plakoglobin, HIC5, Axin, TAK1(MAP3K7), SOX9, Frizzled, DACT1
**7**	Development_NOTCH signaling activation	2.61E-17	NOTCH1 (NICD), CBP, SgK223, NOTCH1 receptor, ADAM12, FRYL, SMRT, MAML1, O-fucose, BRG1, KDELC2, BAF180, MAML2, LSD1, p300, NOTCH2 receptor, DDX5, DLL4, NOTCH2 (2ICD), NOTCH3(NEXT), NOTCH3 (3ICD), CARM1, SKIP (Ski-interacting protein), PHF8, TM4SF9, Itch, NOTCH1 precursor, NOTCH2(NEXT), N-CoR, KDELC1, HDAC1, ZFP64, RBP-J kappa (CBF1), AAK1, NOTCH1 (NEXT), Furin, ADAM10, Jagged1, ADAM17, NUMB, NOTCH3 receptor, Thrombospondin 2
**8**	Development_Positive regulation of WNT/Beta-catenin signaling in the cytoplasm	3.31E-16	Casein kinase II, alpha chains, TBL1X, HECTD1, ITGB1, Bcl-9, LRP5/LRP6, BIG1, SIAH2, IRS-1, Tcf(Lef), 14-3-3 zeta/delta, Alpha-1 catenin, c-Jun, 14-3-3, HSP105, PKA-reg type II (cAMP-dependent), SMAD4, Makorin-1, Dsh, USP47, Trabid, Tankyrases, BIG2, Miz-1, USP9X, USP25, Insulin receptor, USP7, FAK1, GSK3 alpha/beta, IGF-1 receptor, TBLR1, SMAD3, ERK2 (MAPK1), AKT(PKB), Axin, PKA-cat (cAMP-dependent), Frizzled, DACT1
**9**	Transcription_Sin3 and NuRD in transcription regulation	2.56E-15	Mi-2, PPAR-alpha/RXR-alpha, Sin3A, SMRT, SAP130, PPAR-alpha, RXRA, RAR-alpha/RXR-alpha, MTA3, MTA2, ARID4A, MBD2, HDAC2, NRSF, Sin3B, RARalpha, SDS3, Mi-2 alpha, N-CoR, ARID4B, HDAC1, MBD3, NRB54, p66beta, PSF, p66alpha, MTA1
**10**	DNA damage_ATR activation by DNA damage	2.85E-15	NFBD1, HUWE1, LARG, ERK1/2, PRMT5, ATR, p300, TOPBP1, ATRIP, HSP90 beta, p90Rsk, p53BP1, Kendrin, Chk1, Sirtuin1, Miz-1, TTI1, USP7, NMP200, ATM, NEK1, TELO2, Microcephalin, ETAA1, FANCM, USP20 (VDU2), Claspin, Nibrin, TLK1, SNIP1, DBC1
**11**	Transcription_Epigenetic regulation of gene expression	5.17E-15	DNMT3B, HDAC9, GASC1, CBP, SETDB1, PLU-1, MOZ, UTX, HDAC4, PRMT5, DNMT3A, HDAC6, SMCX, LSD1, JMJD1A, p300, DOT1, RBB2, HBOA, MLL1 (HRX), CARM1, Sirtuin1, HDAC2, HDAC8, DNMT1, SET8, HDAC1, MORF, JMJD3, AOF1, Suv39H1, G9a
**12**	Oxidative stress_ROS signaling	6.59E-15	Casein kinase II, alpha chains, NOTCH1 (NICD), p38alpha (MAPK14), Tuberin, ACACA, RelA (p65 NF-kB subunit), SREBP1 (nuclear), KEAP1, IRP1, PKA-cat alpha, ERK1/2, EGR1, PKA-reg (cAMP-dependent), p300, FASN, ELAVL1 (HuR), HSF1, NOTCH3 (3ICD), PRKD1, Sirtuin1, GSK3 beta, p70 S6 kinase1, PKC,

			LKB1, ATM, IKK-beta, DLC1 (Dynein LC8a), IRP2, GRP75, c-Abl, SENP1, Cyclin D1, HIF-prolyl hydroxylase, HDAC1, NF-kB, FIH-1, SAE2, AKT(PKB), SP1, NF-kB p50/p65, p53, c-Src, ADAM17, p38 MAPK, Suv39H1, mTOR
**13**	Regulation of metabolism_Insulin signaling through PI3K/AKT	7.22E-15	Tuberin, eEF2, AS250, ACACA, PI3K cat class IA, AKT1, Raptor, KIAA0528, SREBP1 (nuclear), eIF2B5, GAB1, IRS-1, LAMB1, G6PT, GARNL1, RALGAPB, Rab-8, 14-3-3, eEF2K, Hamartin, FASN, Fibronectin, SREBP1 precursor, p70 S6 kinases, KIF3B, GSK3 beta, p70 S6 kinase1, PKC-lambda/iota, PLC-gamma 1, Insulin receptor, Rab-14, AKT3, DGK, AKT(PKB), Syntaxin 4, PDK (PDPK1), RalA, mTOR
**14**	DNA damage_p53 activation by DNA damage	9.78E-15	p38alpha (MAPK14), CBP, USP10, RelA (p65 NF-kB subunit), Brca1, SMG1, SIAH2, DNA-PK, DAXX, 14-3-3, CABIN1, PP2A regulatory, DDB1, AATF (Che-1), MARKK, ATR, p300, DYRK2, ELAVL1 (HuR), USP11, Chk1, Sirtuin1, Cullin 4A, PP2A structural, USP7, ATM, B56G, c-Abl, COP1, Brca1/Bard1, p53, p38 MAPK, DBC1, PP2C gamma, RFWD3
**15**	Signal transduction_FAK1 signaling	9.81E-15	TRIO, ITGB1, p190-RhoGEF, PI3K cat class IA, KLF8, DOCK1, c-Jun, ERK1/2, RAS, CRK, c-Raf-1, alpha-1/beta-1 integrin, SOS, G-protein alpha-q/11, Fibronectin, Paxillin, p130CAS, GRP-R, PIPKI gamma, MBD2, Talin-1, PDZ-RhoGEF, PLC-beta, ETS, PLC-gamma 1, MMP-14, FAK1, IGF-1 receptor, CtBP, Cyclin D1, HDAC1, AKT(PKB), Collagen IV, PDGF receptor, p53, c-Src, Endophilin A2
**16**	DNA damage_ATM/ATR regulation of G1/S checkpoint	1.68E-13	p38alpha (MAPK14), NFBD1, Brca1, SMG1, FBXW7, beta-TrCP, ERK1/2, PP2A regulatory, CDC25A, ATR, CDC27, CDK2, ELAVL1 (HuR), p70 S6 kinases, Chk1, Cyclin E, PP2A structural, ATM, FOXO3A, B56G, c-Abl, Cyclin D1, Brca1/Bard1, p53, FBXW11, RFWD3
**17**	Cell cycle_DNA replication initiation	2.9E-13	MCM3, RIF1, MCMBP, MCM complex, ORC1L, MCM5, MCM4, MCM10, Jade-1, Importin (karyopherin)-alpha, SSRP1, POLA1, TOPBP1, CDK2, WDHD1, CDC18L (CDC6), HBOA, MCM7, SLD5, Cyclin E, Treslin, RecQL4, MCM6, TRF2, SMARCA5, RPA1, MCM4/6/7 complex, MCM2, GRWD1, Cdt1, HP1 alpha, SUPT16H, RFWD3
**18**	Cell cycle_Senescence activation pathways	4.62E-13	Tuberin, CBP, PI3K cat class IA, MEK3(MAP2K3), ERK1/2, c-Raf-1, SMAD4, ATR, p300, SMAD2, p90Rsk, CDK2, CDK6, Cyclin D2, USP11, Chk1, Sirtuin1, Cyclin E, ATM, FOXO3A, Cyclin D1, SMAD3, AKT(PKB), SP1, HSP90, p53, PDK (PDPK1), p38 MAPK, mTOR
**19**	Signal transduction_Angiotensin II/AGTR1 signaling via	1.79E-12	NOTCH1 (NICD), CBP, RelA (p65 NF-kB subunit), NOTCH1 receptor, TCF7L2 (TCF4), ROCK1, ERK1/2, p300, Fibronectin, NOTCH3(NEXT), NOTCH3 (3ICD), PRKD1, GSK3 beta, PKC, PLC-gamma 1, IKK-beta, IKK-gamma, IGF-1 receptor, Cyclin D1, NCOA1 (SRC1), NF-kB, ERK2 (MAPK1), AKT(PKB), NF-

	Notch, Beta-catenin and NF-kB pathways		kB p50/p65, RBP-J kappa (CBF1), NOTCH1 (NEXT), PDK (PDPK1), ADAM10, c-Myc, ADAM17, p38 MAPK, TAK1(MAP3K7), PKA-cat (cAMP-dependent), mTOR, NOTCH3 receptor
**20**	Transcription_Role of heterochromatin protein 1 (HP1) family in transcriptional silencing	1.85E-12	HDAC9, Mi-2, SETDB1, MeCP2, Sin3A, SUMO-1, HDAC4, CDC25A, DNMT3A, MEF2, HDAC5, HP1 beta, HP1, TIF1-beta, MBD2, HDAC2, Cyclin E, Mi-2 alpha, CtBP, DNMT1, HDAC1, MBD3, HP1 alpha, Suv39H1
**21**	Development_EGFR signaling	1.92E-12	NOTCH1 (NICD), ITGB1, PI3K cat class IA, NOTCH1 receptor, GAB1, JAK1, c-Cbl, c-Jun, ERK1/2, EGR1, CRK, c-Raf-1, SOS, SOS1, p90Rsk, Paxillin, CDK2, STAT3, p130CAS, AP-1, GSK3 beta, p70 S6 kinase1, SOS2, Cyclin E, SHP-2, RIPK1, FAK1, Cyclin D1, NF-kB, ERK2 (MAPK1), AKT(PKB), NOTCH1 (NEXT), c-Src, c-Myc, Mcl-1, mTOR
**22**	Signal transduction_IGF-1 receptor signaling	8.61E-12	Tuberin, NRF1, PI3K cat class IA, RelA (p65 NF-kB subunit), GRP78, Brca1, SREBP1 (nuclear), GAB1, JAK1, IRS-1, G-protein alpha-i family, 14-3-3 zeta/delta, ERK1/2, 14-3-3 gamma, EGR1, c-Raf-1, SOS, p300, MKK7 (MAP2K7), FASN, PKC-epsilon, p90Rsk, STAT3, NF-AT4(NFATC3), GSK3 beta, p70 S6 kinase1, PLC-gamma 1, SHP-2, HMDH, FOXO3A, MMP-14, FAK1, IGF-1 receptor, MNK2(GPRK7), Cyclin D1, SMAD3, AKT(PKB), PDK (PDPK1), Bim, LRP1, mTOR
**23**	Cell cycle_DNA replication: elongation and termination	1.75E-11	POLE1, MCM3, RAD18, TOP1, MCM5, MCM4, VCP, FEN1, POLD cat (p125), CDK2, WDHD1, Chk1, UBE1, MCM7, ATAD5, CHTF18, SHPRH, POLD reg (p68), TOP2 alpha, Ribonuclease H1, MCM6, RFC1, UBC3B, UBE2G2, TIM, MCM2, UBE2G1, Claspin, SMARCA3, DCC1
**24**	Protein folding and maturation_Amyloid precursor protein processing (schema)	1.99E-11	APP-P3, alphaAPPs, BACE1, APP-C99, etaAPP alpha, APP, APP-C31, APP-CTF delta-short, APP-CTF delta-long, betaAPPs, deltaAPPs-80kD, etaAPP beta, APP-CTF theta, APP-C83 (CTF), APP-NCas, Amyloid beta 40, APP-CTF eta, thetaAPPs, Amyloid beta, ADAM10, etaAPPs, ADAM17, APP-C59 (AICD), deltaAPPs-130kD, APP-Jcasp, Amyloid beta 42
**25**	Development_The role of GDNF ligand family/ RET receptor in cell survival, growth and proliferation	2.3E-11	p38alpha (MAPK14), ITGB1, PI3K cat class IA, B-Raf, GAB1, IRS-1, XIAP, PSPN, c-Jun, ERK1/2, EGR1, VEGFR-1, p90RSK2(RPS6KA3), CRK, c-Raf-1, SOS, LIMK1, C3G, Paxillin, CDK2, STAT3, ROCK, PLC-gamma 1, SHP-2, N-Ras, IKK-beta, VAV-2, FAK1, IKK-gamma, CREB1, Cyclin D1, NF-kB, c-FLIP, AKT(PKB), PDK (PDPK1), ARTN, c-Src
**26**	DNA damage_ATM/ATR regulation of G2/M checkpoint: nuclear signaling	3.12E-11	NFBD1, Brca1, PALB2, GTSE1, DNA-PK, CDH1, HDAC6, ATR, CDC14b, ATRIP, p53BP1, SMAR1, CDK2, WDHD1, CDC18L (CDC6), HSF1, Chk1, Wee1, ATM, DNMT1, p53, Mcl-1, Claspin, CEP164

**27**	Chemotaxis_Lysophosphatidic acid signaling via GPCRs	7.03E-11	cPKC (conventional), AKT1, PI3K cat class IA (p110-beta), Tcf(Lef), G-protein alpha-i family, LARG, ROCK1, c-Jun, ERK1/2, PRK1, EGR1, CRK, c-Raf-1, G-protein alpha-q/11, PKC-epsilon, LIMK, Paxillin, MLCP (reg), p130CAS, ROCK, PRKD1, AP-1, GSK3 beta, PAK, p70 S6 kinase1, PDZ-RhoGEF, PKC, PLC-beta, Vinculin, PLEKHG2, DIA1, FAK1, CREB1, Rho GTPase, N-CoR, AKT(PKB), p53, PDK (PDPK1), c-Src, PLC-beta3, ADAM17, PLC-epsilon, p38 MAPK, SRF, mTOR
**28**	DNA damage_Nucleotide excision repair	7.34E-11	Arkadia, Dicer, PARP-1, DTL (hCdt2), Ash1, PIAS4, XPC, VCP, POLR2A, WHSC1, XPF, ATR, DOT1, NEDD4, UBXD7, HIRA, OTUD4, XRCC1, Usp24, DNA polymerase kappa, RAD23B, Cullin 4A, MPP11, DNA ligase III, USP7, RFC1, XPG, Brca1/Bard1, HERC2, SUPT16H
**29**	Development_Positive regulation of STK3/4 (Hippo) pathway and negative regulation of YAP/TAZ function	7.86E-11	Willin, Casein kinase I delta, SCRIB, PKA-cat alpha, STK3, beta-TrCP, Alpha-1 catenin, 14-3-3, PKA-reg (cAMP-dependent), MARKK, c-Raf-1, MARK1, Alpha-catenin, RhoGDI alpha, MOBKL1A, WW45, LATS1, Schwannomin (NF2), LKB1, STK4, Adenylate cyclase, CCDC85C, Itch, MARK4, LATS2, Skp2/TrCP/FBXW, PEZ, AMOTL1 (Jeap), Axin, Ajuba, PKA-cat (cAMP-dependent)
**30**	Proteolysis_SUMOylation pathway	8.57E-11	SUMO-1, PIAS4, TRAF7, SAE1/2, ZNF451, TRIM33, HDAC4, PIAS1, BTBD12, SENP2, SAE1, RanBP2, PIAS3, TIF1-beta, HDAC2, SENP5, TOPORS, RFP, TRIM11, SENP1, SAE2, ZBED1
**31**	DNA damage_G2 checkpoint in response to DNA mismatches	1.02E-10	MutSalpha complex, Brca1, SMC1, MSH3, PMS2, EXO1, ATR, TOPBP1, MSH6, MutSbeta complex, ATRIP, Chk1, MRE11, MSH2, ATM, c-Abl, p53, PMS1, Claspin
**32**	Signal transduction_mTORC2 downstream signaling	1.16E-10	Tuberin, cPKC (conventional), ACACA, OSR1, AKT1, SREBP1 (nuclear), IRS-1, G6PT, PKA-reg (cAMP-dependent), FASN, Adenylate cyclase type IX, FBX29, Paxillin, GSK3 beta, PKC, STK4, PKC-alpha, Insulin receptor, FOXO3A, GSK3 alpha/beta, IGF-1 receptor, Cyclin D1, Filamin A, AKT(PKB), p53, c-Myc, Mcl-1, Bim, PKA-cat (cAMP-dependent), mTOR
**33**	Development_Non-canonical NOTCH signaling	1.24E-10	NOTCH1 (NICD), PI3K cat class IA, AKT1, Cullin 5, RelA (p65 NF-kB subunit), NOTCH1 receptor, E2A, XIAP, MEK3(MAP2K3), DLL4, Cullin 1, STAT3, NOTCH3 (3ICD), GSK3 beta, ATM, IKK-beta, FOXO3A, IKK-gamma, STAT5, Cyclin D1, Mitofusin 2, Bcl-10, c-FLIP, AKT(PKB), p53, PDK (PDPK1), Jagged1, c-Myc, YY1, p38 MAPK, mTOR, NOTCH3 receptor
**34**	Androgen receptor activation and downstream signaling in Prostate cancer	1.44E-10	N-cadherin, B-Raf, GAB1, JAK1, K-RAS, c-Cbl, GCR, NCOA3 (pCIP/SRC3), PI3K cat class IA (p110-beta), IRS-1, Versican, c-Raf-1, SOS, FEN1, APP, SPRY1, NCOA2 (GRIP1/TIF2), Elk-4, FGFR1, STAT3, SHP-2,

			N-Ras, PELP1, Caspase-2, SCAP, IGF-1 receptor, Cyclin D1, NCOA1 (SRC1), PAR1, c-FLIP, ERK2 (MAPK1), AKT(PKB), p53, PDK (PDPK1), ADAM10, ER81, c-Src, c-Myc, ADAM17, ERK1 (MAPK3)
**35**	Development_Negative regulation of WNT/Beta-catenin signaling in the cytoplasm	1.83E-10	KLHL12, HECTD1, LRP5/LRP6, Casein kinase I delta, NOTCH1 receptor, c-Cbl, STK3, Tcf(Lef), HUWE1, Amer1, beta-TrCP, Alpha-1 catenin, PI3K cat class III (Vps34), SENP2, Dsh, Casein kinase I alpha, CDK6, ELAVL1 (HuR), WWP1, LATS1, STK4, PKC-alpha, Itch, Beclin 1, GSK3 alpha/beta, LATS2, WDR26, Skp2/TrCP/FBXW, Cyclin D1, p53, Axin, Frizzled, DACT1
**36**	Transcription_Sirtuin6 regulation and functions	2.46E-10	NRF1, ACACA, USP10, RelA (p65 NF-kB subunit), PARP-1, SREBP1 (nuclear), G6PT, DNA-PK, ELOVL6, S2P, G3BP1 (hdhVIII), SREBP2 (nuclear), FASN, SREBP1 (Golgi membrane), SREBP2 precursor, GPIAP1, S1P, SREBP1 precursor, CPT-1A, Sirtuin1, HMGCS1, LKB1, LDLR, HMDH, SREBP2 (Golgi membrane), FOXO3A, SCAP, SMARCA5
**37**	Development_WNT/Beta-catenin signaling in the nucleus	2.46E-10	LRP5/LRP6, TRRAP, TNIK, Tcf(Lef), AF-10, TCF7L2 (TCF4), c-Jun, BRG1, MED1, MED12, PYGO2, BCL9/B9L, MEF2, TLE, DOT1, CBP/P300, Kaiso, Dsh, UBR5, Mitotic cohesin complex, TCF7L1 (TCF3), SET8, SMARCA5, HDAC1, EP400, NIPBL, p38 MAPK, Frizzled
**38**	DNA damage_ATM/ATR regulation of G2/M checkpoint: cytoplasmic signaling	2.54E-10	p38alpha (MAPK14), Brca1, beta-TrCP, MEK3(MAP2K3), CDC25B, 14-3-3, 14-3-3 gamma, PP2A regulatory, hnRNP A0, MARKK, CDC25A, ATR, Aurora-A, MLCP (reg), TAO2, Chk1, MAPKAPK2, PARN, ATM, FOXO3A, B56G, c-Abl, Brca1/Bard1, ERK2 (MAPK1), p38 MAPK
**39**	Signal transduction_Ephrin-B signaling	3.44E-10	Ephrin-B1, ABL2, p190-RhoGEF, HGK(MAP4K4), Ephrin-B receptor 4, B-Raf, AF-6, ERK1/2, CRK, Ephrin-B receptor 1, Intersectin, p130CAS, ROCK, p120GAP, PAK, Ephrin-B, N-WASP, Ephrin-B receptor 2, FAK1, c-Abl, c-Src, Ephrin-B2, Ephrin-B receptors, NUMB
**40**	Cell adhesion_Integrin-mediated cell adhesion and migration	3.9E-10	Tensin, ITGB1, p190-RhoGEF, PARD3, alpha-4/beta-1 integrin, 14-3-3 zeta/delta, DOCK1, LARG, Alpha-actinin, CRK, alpha-1/beta-1 integrin, Laminin 11, Fibronectin, Paxillin, Talin, p130CAS, PKC, ITGA4, Vinculin, PKC-lambda/iota, Laminin 10, VAV-2, FAK1, Alpha-parvin, GIT2, Collagen IV, c-Src, PARD6
**41**	Signal transduction_Calcium-mediated signaling	6.39E-10	cPKC (conventional), MARK2, RelA (p65 NF-kB subunit), G6PT, MEK3(MAP2K3), c-Jun, ERK1/2, 14-3-3, CABIN1, EGR1, HDAC4, MEF2, HDAC5, p300, MUNC13, RhoGDI alpha, MLCP (reg), ROCK, PKC, PKC-alpha, TORC2, CaMKK, IKK-beta, CREB1, Bcl-10, NF-kB, AKT(PKB), p38 MAPK, SRF, CaMKK2
**42**	Signal transduction_FGFR4 signaling	1.27E-09	PI3K cat class IA, STK3, ETS1, G6PT, c-Jun, ERK1/2, EGR1, RAS, c-Raf-1, PI3K cat class III (Vps34), SOS1, SREBP2 (nuclear), CYP51A1, p130CAS, WW45, ACLY, Schwannomin (NF2), PKC, ERG1, LDLR, SHP-2, HMDH, IKK-beta, PLC-gamma, Beclin 1, SCAP, Nestin, NF-kB, AKT(PKB), SFK, SHIP2, mTOR

**43**	G-protein signaling_RhoB activation	1.31E-09	KIF13A, p190-RhoGEF, GCR, SMURF1, LARG, c-Jun, ERK1/2, PIAS1, ATR, p300, NFYA, ELAVL1 (HuR), Chk1, PDZ-RhoGEF, ARHGEF2, GGTase-I, ATM, FOXO3A, VAV-2, ARF6, SMAD3, TRIF (TICAM1), AKT(PKB), ECT2, p38 MAPK, PDGF-R-beta
**44**	Neurogenesis_NGF/ TrkA MAPK-mediated signaling	1.94E-09	ERK5 (MAPK7), B-Raf, NF-kB1 (p50), K-RAS, MEK3(MAP2K3), c-Jun, ERK1/2, CrkL, EGR1, PP2A regulatory, PKA-reg (cAMP-dependent), CRK, c-Raf-1, SOS, C3G, PKC-epsilon, Ephrin-A receptor 2, p90Rsk, p130CAS, AP-1, MAPKAPK2, PKC-lambda/iota, PLC-gamma 1, SHP-2, N-Ras, KIDINS220, CREB1, Cyclin D1, SP1, PVR, JMJD3, PDZ-GEF1, p107, c-Src, p38 MAPK, PKA-cat (cAMP-dependent), SRF
**45**	Immune response_M-CSF-receptor signaling	1.96E-09	PI3K cat class IA, ERK5 (MAPK7), JAK1, c-Cbl, Tyk2, FMIP, Tcf(Lef), ETS1, c-Jun, ERK1/2, YES, CRK, c-Raf-1, SOS1, STAT5B, STAT3, Cyclin D2, p120GAP, AP-1, MAP3K2 (MEKK2), PKC, PLC-gamma, Fyn, Cyclin D1, NF-kB, AKT(PKB), PDK (PDPK1), c-Src, c-Myc, TSAD, SRF
**46**	DNA damage_ATM activation by DNA damage	2.15E-09	Casein kinase II, alpha chains, NFBD1, Brca1, PARP-1, BRAT1, INTS3, PP2A regulatory, p90RSK2(RPS6KA3), HP1 beta, HSP90 beta, HMG14, MRE11, HDAC2, TTI1, ATM, Itch, Casein kinase II, alpha chain (CSNK2A1), FOXO3A, RNF8, c-Abl, ASCIZ, TELO2, RecQL4, HDAC1, CHFR, HSP90, Nibrin, Suv39H1, NDR1 (STK38)
**47**	Ligand-independent activation of Androgen receptor in Prostate Cancer	2.19E-09	PI3K cat class IA, B-Raf, GAB1, K-RAS, NCOA3 (pCIP/SRC3), IRS-1, Tcf(Lef), PP2A regulatory, c-Raf-1, SOS, NCOA2 (GRIP1/TIF2), DDX5, STAT5B, FGFR1, STAT3, GSK3 beta, N-Ras, c-Abl, IGF-1 receptor, Cyclin D1, NCOA1 (SRC1), HDAC1, ERK2 (MAPK1), AKT(PKB), PDK (PDPK1), c-Myc, ERK1 (MAPK3), Frizzled
**48**	Signal transduction_Additional pathways of NF-kB activation (in the cytoplasm)	2.74E-09	PI3K cat class IA, AKT1, RelA (p65 NF-kB subunit), NF-kB1 (p50), PKA-cat alpha, ERK1/2, PKA-reg (cAMP-dependent), c-Raf-1, PKC-epsilon, CDK6, PRKD1, MAP3K2 (MEKK2), PKC-alpha, PKC-lambda/iota, MAP3K3, Adenylate cyclase, IKK-beta, Casein kinase II, alpha chain (CSNK2A1), IKK-gamma, AKT(PKB), NF-kB p50/p65, PDK (PDPK1), c-Src, TAK1(MAP3K7)
**49**	Development_VEGF signaling via VEGFR2 - generic cascades	2.92E-09	PI3K cat class IA, PAK2, MEK3(MAP2K3), TCF7L2 (TCF4), ROCK1, c-Jun, ERK1/2, c-Raf-1, VEGFR-2, SOS, p90Rsk, Paxillin, p120GAP, GSK3 beta, MAPKAPK2, PKC, Vinculin, PKC-alpha, PLC-gamma 1, IKK-beta, FAK1, IKK-gamma, CREB1, Neurofibromin, Fyn, ERK2 (MAPK1), AKT(PKB), HSP90, NF-kB p50/p65, PDK (PDPK1), c-Src, TSAD, p38 MAPK, ERK1 (MAPK3)
**50**	Transport_Induction of Macropinocytosis	3.02E-09	TRIO, Alpha-actinin 4, PI3K cat class IA, ELMO2, G-protein alpha-i family, 14-3-3 zeta/delta, DOCK1, HDAC6, ALS2, ANKFY1, GGA3, RhoGDI alpha, Rab-5A, PKC, PLC-gamma 1, Insulin receptor, RNTRE,

			PLC-gamma, DLC1 (Dynein LC8a), ARF6, AKT(PKB), HSP90, PDGF receptor, PDK (PDPK1), SHIP2, c-Src, PLC-beta3, CtBP1, WASF2, PDGF-R-beta

Supplementary Table S2: Pathway analysis of genes co-expressed *EXT2* co-expressed genes in gliomas TCGA databases using the MetaCore database (with p-value < 0.05 set as the cutoff value).

#	Maps	pValue	Network Objects from Active Data
**1**	Cytoskeleton remodeling_Regulation of actin cytoskeleton organization by the kinase effectors of Rho GTPases	2.27E-19	MLCP (cat), RhoA, RhoJ, Destrin, F-Actin cytoskeleton, Spectrin, SLC9A1, PRK1, Myosin II, Caldesmon, Alpha-actinin, RhoC, LIMK1, PIP5KI, MyHC, LIMK, Paxillin, Rac1, MRCK, RhoGDI alpha, MLCP (reg), ROCK, Actomyosin, MRCKalpha, PAK, Cofilin, non-muscle, Rac1-related, Cdc42 subfamily, Vinculin, MSN (moesin), ERM proteins, MRLC, RhoA-related, CDC42, Actin cytoskeletal, Filamin A, Cofilin
**2**	Chemotaxis_Lysophosphatidic acid signaling via GPCRs	5.14E-19	RhoA, cPKC (conventional), G-protein alpha-12 family, AKT1, Tcf(Lef), G-protein alpha-i family, H-Ras, TRAF6, F-Actin cytoskeleton, LARG, ROCK1, c-Jun, PRK1, HB-EGF, Beta-catenin, EGR1, CRK, G-protein alpha-q/11, p21, LIMK, TRIP6, Paxillin, Rac1, IP3 receptor, YAP1 (YAp65), ATF-2/c-Jun, G-protein gamma 12, MLCP (reg), ROCK, PRKD1, Bax, AP-1, Caspase-7, PAK, p70 S6 kinase1, PKC, FKHR, Vinculin, ATF-2, DIA1, Caspase-3, Cyr61, CREB1, Bcl-XL, MEK1/2, G-protein beta/gamma, Rho GTPase, CDC42, Actin cytoskeletal, N-CoR, TAZ, CTGF, AKT(PKB), p53, ADAM17, p38 MAPK, SRF, Cofilin
**3**	Protein folding and maturation_Amyloid precursor protein processing (schema)	6.3E-16	Caspase-6, APP-P3, alphaAPPs, BACE1, APP-C99, etaAPP alpha, APP, Caspase-8, APP-C31, APP-CTF delta-short, APP-CTF delta-long, betaAPPs, ADAM9, deltaAPPs-80kD, etaAPP beta, APP-CTF theta, Caspase-3, BACE2, APP-C83 (CTF), APP-NCas, Amyloid beta 40, APP-CTF eta, thetaAPPs, Amyloid beta, ADAM10, etaAPPs, ADAM17, APP-C59 (AICD), deltaAPPs-130kD, APP-Jcasp, Amyloid beta 42
**4**	Oxidative stress_ROS signaling	1.96E-14	Casein kinase II, alpha chains, p38alpha (MAPK14), Thioredoxin, RelA (p65 NF-kB subunit), iNOS, KEAP1, Adrenomedullin, IRP1, PKA-cat alpha, HIF1A, AMPK alpha subunit, EGR1, PKA-reg (cAMP-dependent), Isoform p66 Shc, p21, Bak, NFKBIA, GSTP1, TfR1, ELAVL1 (HuR), NOTCH3 (3ICD), PRKD1, Bax, p70 S6 kinase1, PKC, LKB1, DLC1 (Dynein LC8a), IRP2, GRP75, c-Abl,

			SENP1, GADD45 alpha, HIF-prolyl hydroxylase, HDAC1, NF-kB, SAE2, AKT(PKB), SP1, Catalase, NF-kB p50/p65, p53, NRF2, ADAM17, p38 MAPK, APEX, PAI1
**5**	Development_The role of GDNF ligand family/ RET receptor in cell survival, growth and proliferation	4.38E-13	RhoA, p38alpha (MAPK14), ITGB1, PI3K cat class IA, NCK1, HIF1A, H-Ras, XIAP, F-Actin cytoskeleton, ATF-1, c-Jun, EGR1, p90RSK2(RPS6KA3), CRK, SOS, LIMK1, NFKBIA, Paxillin, Rac1, IP3 receptor, CDK2, STAT3, RAP-1A, GDNF, Shc, ROCK, Cyclin A2, N-Ras, VAV-2, IKK-gamma, CREB1, Bcl-XL, MEK1/2, DOK1, CDC42, NF-kB, c-FLIP, AKT(PKB), ARTN, Cofilin
**6**	Tau dysregulation in Alzheimer disease	5.79E-13	G3P2, Calcineurin A (alpha), Casein kinase I delta, AKT1, Calcineurin A (catalytic), HSC70, Caspase-6, C/EBPbeta, PP1-cat, SET, PP2A regulatory, PP2A catalytic, PICALM, Calcipressin 1, APP, PP2A cat (alpha), Caspase-8, PPP2R5E, Calpain 2(m), RanBPM, FKBP5, p70 S6 kinases, RBBP7 (RbAp46), CIP2A, Caspase-3, c-Abl, Caspase-2, Fyn, TARDBP (TDP43), PRNP, AMPK alpha 1 subunit, AKT(PKB), PPME1, HSP90, Calpain 1(mu), APP-C59 (AICD), PKA-cat (cAMP-dependent), Amyloid beta 42
**7**	Signal transduction_S1P2 receptor activation signaling	1.55E-12	MLCP (cat), RhoA, SMAD5, G-protein alpha-12 family, PI3K cat class IA, BMP receptor 2, ICAM1, G-protein alpha-12, G-protein alpha-i family, H-Ras, LARG, ROCK1, c-Jun, HB-EGF, Beta-catenin, EGR1, SMAD4, LIMK1, G-protein alpha-i2, NFKBIA, Paxillin, IP3 receptor, YAP1 (YAp65), G-protein alpha-13, STAT3, MLCP (reg), ROCK, AP-1, MRLC, LIF, CREB1, MEK1/2, G-protein beta/gamma, Actin cytoskeletal, G-protein alpha-q, SMAD1, NF-kB, Osteoprotegerin, AKT(PKB), p38 MAPK, SRF, Cofilin, NOTCH3 receptor
**8**	Transcription_HIF-1 targets	1.55E-12	G3P2, ARNT, NIX, iNOS, Lysyl oxidase, ROR-alpha, Adrenomedullin, HIF1A, HXK1, HIF-1, SLC9A1, P4HA1, 5'-NTD, FGF2, GLUT3, Angiopoietin 2, p21, MGF, MSH6, PGK1, AK3, IBP3, TfR1, GLUT1, Carbonic anhydrase XII, MSH2, Nucleophosmin, SOX2, TGF-beta 3, LOXL4, LOXL2, CTGF, LDHA, DEC1 (Stra13), p53, Mcl-1, PAI1, CXCR4, LRP1, PKM2
**9**	Cell adhesion_Integrin-mediated cell adhesion and migration	3.42E-12	RhoA, alpha-3/beta-1 integrin, ITGB1, PARD3, alpha-4/beta-1 integrin, ICAM1, PINCH, 14-3-3 zeta/delta, F-Actin cytoskeleton, LARG, alpha-5/beta-1 integrin, Alpha-actinin, CRK, alpha-1/beta-1 integrin, Fibronectin, Paxillin, Rac1, PKC, ITGA4, Vinculin, PKC-lambda/iota, VASP, Zyxin, VAV-2, alpha-2/beta-1 integrin, CDC42, Actin cytoskeletal, Alpha-parvin, GIT2, VAV-3, Collagen IV
**10**	Development_Positive regulation of STK3/4 (Hippo) pathway and negative	5.14E-12	RhoA, Casein kinase I delta, MPP5, PKA-cat alpha, STK3, AMPK beta subunit, Alpha-1 catenin, 14-3-3, AMPK alpha subunit, Beta-catenin, PKA-reg (cAMP-dependent), MARKK, PP2A cat (alpha), Actin, Alpha-catenin, YAP1 (YAp65), RhoGDI alpha, WW45, MALS-3, LKB1, STK4, Adenylate cyclase,

	regulation of YAP/TAZ function		Itch, LIF, RASSF5, LATS2, Mol1b, TAZ, PEZ, LRR-1, Ajuba, PKA-cat (cAMP-dependent), LIF receptor
**11**	FAK1 signaling in melanoma	1.63E-11	RhoA, ITGB1, RelA (p65 NF-kB subunit), Syntenin 1, ROCK2, alpha-5/beta-1 integrin, CRK, SOS, RhoC, Fibronectin, NFKBIA, PLAU (UPA), Paxillin, Rac1, Shc, PKC-alpha, N-Ras, MEK1/2, Actin cytoskeletal, NF-kB, CAS-L, Caveolin-1, ITGA5
**12**	Cell cycle_Influence of Ras and Rho proteins on G1/S Transition	3.03E-11	MLCP (cat), MEK1(MAP2K1), RhoA, PI3K cat class IA, RelA (p65 NF-kB subunit), ROCK2, H-Ras, alpha-5/beta-1 integrin, p21, NFKBIA, Rac1, CDK2, CDK6, STAT3, MLCP (reg), p70 S6 kinase1, Rb protein, Cyclin A2, ATF-2, DIA1, Cyclin E, MRLC, RGL2, CDC42, AKT(PKB), NF-kB p50/p65, RalA
**13**	DNA damage_p53 activation by DNA damage	4.23E-11	p38alpha (MAPK14), USP10, RelA (p65 NF-kB subunit), PML, SIAH2, DNA-PK, DAXX, 14-3-3, PP2A regulatory, MARKK, PP2A catalytic, p21, ATR, ELAVL1 (HuR), Tip60, Chk1, Bax, DDB2, Cullin 4A, TTC5 (Strap), USP7, B56G, c-Abl, Bcl-XL, COP1, GADD45 alpha, PIG3, p53, p38 MAPK, PP2C gamma, RFWD3
**14**	Cytoskeleton remodeling_Role of PKA in cytoskeleton reorganization	6.75E-11	MLCP (cat), RhoA, LBC, alpha-4/beta-1 integrin, F-Actin cytoskeleton, PKA-reg (cAMP-dependent), LIMK1, Paxillin, Rac1, IP3 receptor, MLCP (reg), 14-3-3 beta/alpha, ROCK, VASP, Adenylate cyclase, c-Abl, G-protein beta/gamma, CDC42, Actin cytoskeletal, LASP1, MELC, PKA-cat (cAMP-dependent), Cofilin
**15**	Apoptosis and survival_NGF/ TrkA PI3K-mediated signaling	7.83E-11	MLCP (cat), RhoA, PI3K cat class IA, PARD3, AKT1, Calcineurin A (catalytic), H-Ras, Destrin, PAK2, Myosin II, SOS, LIMK1, Rac1, RAP-1A, MLCP (reg), Shc, ROCK, MRCKalpha, p70 S6 kinase1, Cofilin, non-muscle, FKHR, MSN (moesin), N-WASP, MRLC, VAV-2, CREB1, CDC42, Actin cytoskeletal, VAV-3, AKT(PKB), ARAP3, SSH1L, Cofilin
**16**	Development_VEGF signaling via VEGFR2 - generic cascades	7.88E-11	MEK1(MAP2K1), RhoA, PI3K cat class IA, Calcineurin A (catalytic), iNOS, NCK1, H-Ras, PAK2, MEK3(MAP2K3), ROCK1, c-Jun, eIF4E, Beta-catenin, SOS, p90Rsk, PLAU (UPA), Paxillin, Rac1, IP3 receptor, I-kB, Shc, COX-1 (PTGS1), p120GAP, MAPKAPK2, PKC, Vinculin, PKC-alpha, IKK-gamma, CREB1, CDC42, Actin cytoskeletal, Fyn, AKT(PKB), HSP90, NF-kB p50/p65, PLA2G5, p38 MAPK
**17**	Signal transduction_S1P1 receptor signaling	9.69E-11	MEK1(MAP2K1), RhoA, VE-cadherin, AKT1, RelA (p65 NF-kB subunit), ICAM1, KLF5, Tcf(Lef), G-protein alpha-i family, H-Ras, c-Jun, PDGF-A, PI3K class II (CII-alpha), Beta-catenin, Alpha-actinin, CRK, G-protein alpha-i3, SOS, LIMK1, G-protein alpha-i2, Alpha-catenin, Paxillin, Rac1, IP3 receptor,

			YAP1 (YAp65), Shc, p70 S6 kinases, PI3K cat class IA (p110-alpha), AP-1, MAPKAPK2, PKC-alpha, Adenylate cyclase, Cyr61, MMP-14, MEK1/2, G-protein beta/gamma, CDC42, Fyn, CTGF, Osteoprotegerin, AKT(PKB), p38 MAPK, Cofilin
**18**	Signal transduction_ESR1 (membrane) and ESR2 (membrane) signaling	1.49E-10	RhoA, cPKC (conventional), PI3K cat class IA, ROCK2, Tcf(Lef), C/EBPbeta, G-protein alpha-i family, H-Ras, HB-EGF, AMPK alpha subunit, Beta-catenin, EGR1, PKA-reg (cAMP-dependent), G-protein alpha-i3, SOS, ZNF370, G-protein alpha-i2, p90Rsk, ESR2 (membrane), Rac1, IP3 receptor, G-protein alpha-13, Shc, p120GAP, Profilin, PKC, LKB1, PKC-alpha, MSN (moesin), CTH, N-WASP, Adenylate cyclase, CREB1, MEK1/2, G-protein beta/gamma, CDC42, G-protein alpha-q, AKT(PKB), Caveolin-1, PKA-cat (cAMP-dependent), SRF, Cofilin
**19**	Cell adhesion_PLAU signaling	3.41E-10	Casein kinase II, alpha chains, MEK1(MAP2K1), RhoA, alpha-3/beta-1 integrin, c-IAP2, gp130, PI3K cat class IA, alpha-V/beta-1 integrin, G-protein alpha-i family, H-Ras, XIAP, F-Actin cytoskeleton, alpha-5/beta-1 integrin, G-protein alpha-i3, SOS, alpha-V/beta-5 integrin, PLAU (UPA), Paxillin, Rac1, STAT3, Shc, ROCK, Nucleolin, c-IAP1, MRLC, CDC42, NF-kB, AKT(PKB), Caveolin-1
**20**	Signal transduction_Non-neuronal ACM1, ACM3 and ACM5 signaling	5.84E-10	MLCP (cat), RhoA, PI3K cat class IA, iNOS, G-protein alpha-12, H-Ras, c-Jun, G-protein alpha-11, MRLC2, PKA-reg (cAMP-dependent), G-protein alpha-q/11, p21, Rac1, CDK2, MLCP (reg), ROCK, p70 S6 kinases, Rb protein, Adenylate cyclase type VIII, ITGA2, PKC, ITGA3, PKC-alpha, Adenylate cyclase, MRLC, G-protein alpha-q, AKT(PKB), SP1, PKA-cat (cAMP-dependent), SRF, Adenylate cyclase type III
**21**	Apo-2L(TNFSF10)-induced apoptosis in melanoma	8.15E-10	DR5(TNFRSF10B), RelA (p65 NF-kB subunit), NF-kB1 (p50), XBP1, XIAP, Caspase-4, c-FLIP(Long), TRADD, Bak, FADD, Caspase-8, Dynamin-2, NFKBIA, Bax, Caspase-3, RIPK1, IKK-gamma, c-FLIP(Short), NF-kB, IRE1, DR4(TNFRSF10A), NF-kB p50/p65, ATF-6 alpha (50kDa)
**22**	FGF2 signaling in melanoma	1.27E-09	RhoA, PI3K cat class IA, Syndecan-1, Fra-1, Syndecan-4, YES, FGF2, SOS, p21, Fibronectin, FGFR1, Rac1, STAT3, Shc, N-Ras, MEK1/2, CDC42, Caveolin-1, Collagen IV, SPRY2, p38 MAPK
**23**	Immune response_B cell antigen receptor (BCR) pathway	1.36E-09	MEK1(MAP2K1), STIM1, RelA (p65 NF-kB subunit), Calcineurin A (catalytic), NCK1, NF-kB1 (p50), alpha-4/beta-1 integrin, ICAM1, H-Ras, MEK3(MAP2K3), CIN85, EGR1, PP2A catalytic, Fibronectin, PIP5KI, NFKBIA, Rac1, IP3 receptor, CDK6, Shc, ORAI1, p70 S6 kinase1, Rb protein, FKHR, ATF-2, MALT1, N-Ras, VAV-2, IKK-gamma, Bcl-XL, MEK1/2, CDC42, Actin cytoskeletal, Bcl-10, NF-kB, AKT(PKB), NF-kB p50/p65, p38 MAPK, TAK1(MAP3K7)

**24**	Inhibition of Ephrin receptors in colorectal cancer	1.84E-09	Ephrin-B1, RhoA, Ephrin-B receptor 4, KLF5, Beta-catenin, Ephrin-A receptors, Ephrin-A receptor 2, Paxillin, Rac1, RAP-1A, ROCK, Ephrin-B, VAV-2, CDC42, Ephrin-A, Ephrin-B2, Ephrin-B receptors, Frizzled
**25**	Apoptosis and survival_TNF-alpha-induced Caspase-8 signaling	1.86E-09	AKT1, Caspase-6, FAN, HSP90 alpha, PP2A regulatory, TRADD, PP2A catalytic, FADD, Caspase-8, Acid sphingomyelinase, Bax, Caspase-7, ErbB2, Caspase-3, RIPK1, Caspase-2, AKT2, c-FLIP(Short), AKT(PKB), HSP90, TNF-R1, ADAM17
**26**	Immune response_Antigen presentation by MHC class I, classical pathway	2.17E-09	PDIA3, MIC2, GANAB, UGCGL1, Endoplasmin, PSMB5, HSP90 alpha, PA28 (11S regulator), Tapasin, Impas 1, PSME1, TAP1 (PSF1), Sec24, PSME2, NPEPPS, Nardilysin, Calreticulin, Calnexin, TAPBPL, Sec23, HSP70, Furin, BCAP31, IDE, ERAP1
**27**	Anti-apoptotic pathways in endoplasmic reticulum stress response in multiple myeloma	2.25E-09	GRP78, eIF2AK3, XBP1, PP1-cat, Endoplasmin, S2P, GADD34, ATF-6 alpha (90kDa), S1P, Calreticulin, eIF2S1, IRE1, p38 MAPK, ATF-6 alpha (50kDa)
**28**	Development_SLIT-ROBO1 signaling	2.36E-09	RhoA, PI3K cat class IA, Calcineurin A (catalytic), NCK1, ROBO1, F-Actin cytoskeleton, PAK2, Myosin II, Rac1, ROCK, FLII, ARF6, CDC42, Fyn, Cytohesin3, AKT(PKB), SSH1L, Endophilin A2, CXCR4, ACTB, Cofilin
**29**	Signal transduction_Non-canonical WNT5A signaling	2.64E-09	MLCP (cat), MEK1(MAP2K1), RhoA, cPKC (conventional), Casein kinase I delta, AKT1, Calcineurin A (catalytic), G-protein alpha-i family, Lef-1, c-Jun, Beta-catenin, FZD3, RYK, Paxillin, Rac1, IP3 receptor, MLCP (reg), ROCK, PKC-lambda/iota, ATF-2, FZD5, LRP5, G-protein beta/gamma, CDC42, G-protein alpha-q, FZD9, Filamin A, SP1, FZD7, p38 MAPK, TAK1(MAP3K7), Cofilin
**30**	Autophagy_Autophagy	2.8E-09	APG3, PI3K reg class III (p150), APG16L1, Raptor, C/EBP zeta, HSC70, KEAP1, eIF2AK3, AMPK alpha subunit, Sec8, PI3K cat class III (Vps34), Syntaxin 17, Tip60, GCN2, RalB, FKHR, PKC-alpha, SOX2, APG5, ATG13, AMBRA1, Beclin 1, CREB1, eIF2S1, NRBF2, p53, Atg101, FIP200
**31**	Stellate cells activation and liver fibrosis	2.85E-09	MEK1(MAP2K1), Biglycan, PI3K cat class IA, ICAM1, Tcf(Lef), H-Ras, TRAF6, IL1RAP, KLF6, Beta-catenin, TRADD, SHH, SOS, SMAD4, SMAD2, SARA, Smoothened, TIMP1, I-kB, Shc, MyD88, RIPK1, IKK-gamma, IRAK1/2, AKT(PKB), SP1, NF-kB p50/p65, TNF-R1, Frizzled
**32**	Role of Apo-2L(TNFSF10) in Prostate Cancer cell apoptosis	3.42E-09	c-IAP2, DR5(TNFRSF10B), XIAP, c-FLIP(Long), TRADD, Bak, FADD, Caspase-8, I-kB, Bax, Caspase-7, c-IAP1, Caspase-3, RIPK1, IKK-gamma, Bcl-XL, NF-kB, Osteoprotegerin, DR4(TNFRSF10A)

**33**	Cytoskeleton remodeling_PDGF signaling via calcium and Rho GTPases	3.77E-09	SLC31A1, N-cadherin, ABL2, STIM1, AKT1, Lysyl oxidase, NCK1, F-Actin, PDGF-A, Beta-catenin, IQGAP1, Dynamin-2, Paxillin, Rac1, IP3 receptor, ORAI1, ARP2, PKC, Vinculin, PKC-alpha, N-WASP, VAV-2, c-Abl, PDGF-C, CDC42, Actin cytoskeletal, Fyn, ALPHA-PIX, PKA-cat (cAMP-dependent), PDGF-D, ARPC2, WaspIP
**34**	Chemotaxis_SDF-1/ CXCR4-induced chemotaxis of immune cells	3.98E-09	RhoA, ITGB1, PI3K cat class IA, NCK1, alpha-4/beta-1 integrin, ICAM1, PKA-cat alpha, G-protein alpha-i family, F-Actin cytoskeleton, PAK2, ROCK1, PKA-reg (cAMP-dependent), CRK, LIMK1, Paxillin, Rac1, G-protein alpha-13, RAP-1A, Shc, PAK, p70 S6 kinase1, Vinculin, RASSF5, MEK1/2, G-protein beta/gamma, CDC42, Fyn, AKT(PKB), SFK, CXCR4, Cofilin
**35**	Signal transduction_S1P2 receptor inhibitory signaling	5.54E-09	MLCP (cat), RhoA, VE-cadherin, G-protein alpha-12 family, G-protein alpha-12, G-protein alpha-i family, H-Ras, LARG, ROCK1, PKA-reg (cAMP-dependent), Alpha-actinin, RhoC, G-protein alpha-i2, Rac1, IP3 receptor, G-protein alpha-13, MLCP (reg), ROCK, Adenylate cyclase, MRLC, MEK1/2, G-protein beta/gamma, G-protein alpha-q, AKT(PKB), PKA-cat (cAMP-dependent)
**36**	Cell adhesion_Histamine H1 receptor signaling in the interruption of cell barrier integrity	5.6E-09	MLCP (cat), RhoA, VE-cadherin, Myosin II, Beta-catenin, Alpha-actinin, G-protein alpha-q/11, LIMK1, Alpha-catenin, Paxillin, IP3 receptor, Histamine H1 receptor, MLCP (reg), ROCK, Vinculin, PKC-alpha, MRLC, G-protein beta/gamma, Actin cytoskeletal, MELC, p120-catenin, Cofilin
**37**	Signal transduction_Calcium-mediated signaling	6.21E-09	MLCP (cat), RhoA, cPKC (conventional), RelA (p65 NF-kB subunit), Calcineurin A (catalytic), G6PT, MEK3(MAP2K3), c-Jun, 14-3-3, CaMK I, AMPK alpha subunit, Myosin II, EGR1, Rac1, IP3 receptor, RhoGDI alpha, MLCP (reg), I-kB, ROCK, PKC, PKC-alpha, ATF-2, MALT1, CREB1, Bcl-10, NF-kB, AKT(PKB), p38 MAPK, SRF
**38**	Neurophysiological process_Melatonin signaling in the nervous system	6.21E-09	RhoA, cPKC (conventional), PI3K cat class IA, RelA (p65 NF-kB subunit), ROR-alpha, C/EBPbeta, G-protein alpha-i family, Beta-catenin, PKA-reg (cAMP-dependent), G-protein alpha-i3, G-protein alpha-q/11, Vimentin, G-protein alpha-i2, p90Rsk, IP3 receptor, 14-3-3 beta/alpha, ROCK, GLUT1, Bax, PKC, PKC-alpha, Adenylate cyclase, CREB1, MEK1/2, G-protein beta/gamma, NF-kB, AKT(PKB), NRF2, PKA-cat (cAMP-dependent)
**39**	Development_Regulation of epithelial-to-mesenchymal transition (EMT)	6.22E-09	VE-cadherin, N-cadherin, Arkadia, RelA (p65 NF-kB subunit), E2A, Lef-1, c-Jun, PDGF-A, Caldesmon, FGF2, TGIF, SMAD2, Fibronectin, Vimentin, FGFR1, TGF-beta 3, ATF-2, SLUG, CREB1, SP1, TNF-R1, Jagged1, PAI1, SRF, PDGF-D, Frizzled, ACTB

**40**	Immune response_Sublytic effects of membrane attack complex	6.3E-09	RhoA, AKT1, GRP78, eIF2AK3, C/EBPbeta, Endoplasmin, G-protein alpha-i family, H-Ras, TRAF6, MEK3(MAP2K3), c-FLIP(Long), MCU, SOS, Caspase-8, IP3 receptor, MAPKAPK2, PKC, GRP75, Bcl-XL, MEK1/2, G-protein beta/gamma, Actin cytoskeletal, eIF2S1, AKT(PKB), Pnpla8, p38 MAPK, TAK1(MAP3K7), ATF-6 alpha (50kDa)
**41**	Development_Regulation of telomere length and cellular immortalization	6.58E-09	hnRNP A1, PI3K cat class IA, Staufen, TIN-2, hnRNP C, Dyskerin (NAP57), Ku80, TRF1, PKC-alpha, Ku70/80, PTOP, Ku70, POT1, Max, AKT(PKB), SP1, HSP90, p23 co-chaperone, Tankyrase 1
**42**	TGF-beta 1-induced transactivation of membrane receptors signaling in hepatocellular carcinoma (HCC)	1.11E-08	alpha-3/beta-1 integrin, ITGB1, PI3K cat class IA, Lef-1, PDGF-A, Beta-catenin, alpha-5/beta-1 integrin, TGF-beta, CRK, LIMK1, Fibronectin, Actin, Rac1, CDK2, ITGA2, Cyclin E, SLUG, c-Abl, alpha-2/beta-1 integrin, Cyclin A, AKT(PKB), ITGA5, Cofilin
**43**	Development_EGFR signaling	1.14E-08	ITGB1, PI3K cat class IA, NCK1, H-Ras, c-Jun, HB-EGF, EGR1, FGF2, CRK, SOS, p90Rsk, NFKBIA, Paxillin, CDK2, STAT3, Shc, EGR2 (Krox20), AP-1, Amphiregulin, p70 S6 kinase1, ErbB2, SOS2, Cyclin E, Caspase-3, RIPK1, MEK1/2, NF-kB, CTGF, AKT(PKB), p120-catenin, Mcl-1
**44**	Resistance of pancreatic cancer cells to death receptor signaling	1.5E-08	c-IAP2, DR5(TNFRSF10B), RelA (p65 NF-kB subunit), Caspase-6, XIAP, c-FLIP(Long), FADD, Caspase-8, NFKBIA, PRKD1, Bax, Caspase-7, FAP-1, c-IAP1, Caspase-3, Bcl-XL, c-FLIP(Short), DR4(TNFRSF10A)
**45**	Brca1 in ovarian cancer	1.92E-08	PML, NF-kB1 (p105), BACH1, XIAP, c-Jun, p21, Ubiquitin, Bax, BRIP1, c-IAP1, Caspase-3, AKT(PKB), RBBP8 (CtIP), p53, Bard1, RAP80
**46**	CFTR folding and maturation (normal and cystic fibrosis)	1.95E-08	MA1B1, OST complex, GANAB, UGCGL1, EDEM, HSP90 alpha, ERp29, Sti1, HSP90 beta, DNAJB6 (Hdj-1), Csp, HSP40, Calnexin, HSP70, p23 co-chaperone
**47**	Development_Stimulation of differentiation of mouse embryonic fibroblasts into adipocytes by extracellular factors	1.97E-08	MEK1(MAP2K1), AKT1, Lysyl oxidase, BMP receptor 2, IRS-2, C/EBPbeta, H-Ras, XIAP, ATF-1, p90RSK2(RPS6KA3), PKA-reg (cAMP-dependent), SOS, SMAD4, Shc, EGR2 (Krox20), PI3K cat class IA (p110-alpha), FKHR, ATF-2, Adenylate cyclase, LIF, CREB1, AKT2, SMAD1, p38 MAPK, PKA-cat (cAMP-dependent), LPL, LIF receptor, C/EBPdelta

**48**	Apoptosis and survival_Endoplasmic reticulum stress response	2.03E-08	GRP78, C/EBP zeta, EDEM, KEAP1, eIF2AK3, XBP1, Endoplasmin, c-Jun, S2P, GADD34, ATF-6 alpha (90kDa), PP2A catalytic, Calpain 2(m), S1P, BTEB1, DNAJB11, EDEM2, Caspase-3, Bcl-XL, eIF2S1, IRE1, DnaJB9, NRF2, Mcl-1, ATF-6 alpha (50kDa)
**49**	Inhibition of remyelination in multiple sclerosis: regulation of cytoskeleton proteins	2.15E-08	MLCP (cat), RhoA, ROCK2, alpha-V/beta-1 integrin, chTOG, Myosin II, TGF-beta, LIMK1, hnRNP A2, Fibronectin, Paxillin, Rac1, MLCP (reg), Tubulin alpha, PKC-alpha, MRLC, CDC42, Actin cytoskeletal, Fyn, MELC, Cofilin
**50**	Signal transduction_Ephrin-B signaling	2.52E-08	Ephrin-B1, RhoA, ABL2, Ephrin-B receptor 4, NCK1, Serine racemase, H-Ras, CRK, Rac1, ROCK, p120GAP, PAK, Ephrin-B, N-WASP, c-Abl, MEK1/2, DOK1, CDC42, Caveolin-1, Ephrin-B2, Ephrin-B receptors, NUMB
